# The *Leishmania* PABP1–eIF4E4 interface: a novel 5′–3′ interaction architecture for trans-spliced mRNAs

**DOI:** 10.1093/nar/gky1187

**Published:** 2018-11-22

**Authors:** Fabio Henrique dos Santos Rodrigues, Helena Firczuk, Alexander L Breeze, Alexander D Cameron, Martin Walko, Andrew J Wilson, Nilson I T Zanchin, John E G McCarthy

**Affiliations:** 1 Warwick Integrative Synthetic Biology Centre (WISB) and School of Life Sciences, University of Warwick, Gibbet Hill, Coventry CV4 7AL, UK; 2 Astbury Centre for Structural Molecular Biology, University of Leeds, LS2 9JT, UK; 3 Faculty of Biological Sciences, University of Leeds, LS2 9JT, UK; 4 School of Chemistry, University of Leeds, LS2 9JT, UK; 5 Instituto Carlos Chagas, FIOCRUZ-Paraná, Rua Professor Algacyr Munhoz Mader 3775, Curitiba, PR 81350-010, Brazil

## Abstract

Trans-splicing of trypanosomatid polycistronic transcripts produces polyadenylated monocistronic mRNAs modified to form the 5′ cap4 structure (m^7^Gpppm_3_^6,6,2′^Apm^2′^Apm^2′^Cpm_2_^3,2′^U). NMR and X-ray crystallography reveal that *Leishmania* has a unique type of N-terminally-extended cap-binding protein (eIF4E4) that binds via a PAM2 motif to PABP1. This relies on the interactions of a combination of polar and charged amino acid side-chains together with multiple hydrophobic interactions, and underpins a novel architecture in the *Leishmania* cap4-binding translation factor complex. Measurements using microscale thermophoresis, fluorescence anisotropy and surface plasmon resonance characterize the key interactions driving assembly of the *Leishmania* translation initiation complex. We demonstrate that this complex can accommodate *Leishmania* eIF4G3 which, unlike the standard eukaryotic initiation complex paradigm, binds tightly to eIF4E4, but not to PABP1. Thus, in *Leishmania*, the chain of interactions 5′cap4-eIF4E4–PABP1-poly(A) bridges the mRNA 5′ and 3′ ends. Exceptionally, therefore, by binding tightly to two protein ligands and to the mRNA 5′ cap4 structure, the trypanosomatid N-terminally extended form of eIF4E acts as the core molecular scaffold for the mRNA-cap-binding complex. Finally, the eIF4E4 N-terminal extension is an intrinsically disordered region that transitions to a partly folded form upon binding to PABP1, whereby this interaction is not modulated by poly(A) binding to PABP1.

## INTRODUCTION

The eukaryotic translation machinery is highly complex, comprising not only ribosomes and tRNAs but also a host of translation factors that promote mRNA recruitment and AUG (start codon) recognition, as well as polypeptide elongation and termination (1,2). While it has been evident for some time that there are variations in terms of the structural and functional properties of eukaryotic translation factors across the animals, plants and fungi (3), recent work has highlighted particularly distinctive features of the trypanosomatid translation machinery. These differences are of special interest, not only in the context of our fundamental understanding of biology, but also because trypanosomatids are a worldwide threat to human health and analysis of distinctive molecular features may help identify potential drug targets. Approximately 37 million people are thought to be infected collectively with *Trypanosoma brucei* (African sleeping sickness), *Trypanosoma cruzi* (Chagas disease) and *Leishmania* species (responsible for multiple forms of leishmaniasis), and many more people are at risk of infection (4). It is striking that trypanosomatids manifest a combination of special cellular and biomolecular attributes; for example, transcription of large polycistronic chromosomal clusters followed by *trans*-splicing and polyadenylation into monocistronic mRNAs (5,6), a pronounced reliance on posttranscriptional control mechanisms (7), and other unusual features (8–10).

One remarkable feature of trypanosomatid translation machineries is the involvement of an exceptionally large number of isomers of both of the translation factors eIF4E [six isomers (11)] and eIF4G [five isomers (7)]. The eIF4E isomers bind to the highly modified cap4 structure (m^7^Gpppm_3_^6,6,2′^Apm^2′^Apm^2′^Cpm_2_^3,2′^U) that is added to the 5′end of the monocistronic mRNAs during processing (12–14), and are thought to fulfill differentiated functions over the *Leishmania* life cycle (15). Another distinctive feature is that two of the eIF4E isomers (3 and 4) are cytoplasmic proteins with long N-terminal extensions that are not evident in the shared architecture of the eIF4E counterparts that have been characterised in animals, plants and fungi (11). It is thought that eIF4E3 and eIF4E4 are the only trypanosomatid eIF4E species likely to underpin general translation and that, of these two isomers, eIF4E4 plays the more significant role (7). Moreover, the N-terminal extension of *Leishmania* eIF4E4 can interact directly with PABP1. PABP1 binding was originally reported to require the first 86 amino acids of an expression construct that had a 140-codon 5′-deletion from the eIF4E4 reading frame (16), while later work indicated that PABP1 binding depends on more central regions of what came to be recognized as the full-length reading frame (11).

Interactions between proteins that associate with the 5′ end and the 3′ end of mRNA have been identified in animals, plants and fungi. Up until now, research in this area has focused on the role of eIF4G as a bridge between the cap-binding protein eIF4E and the poly(A) binding protein PABP (17). This is thought to be related to the observation that mRNAs that are both capped and polyadenylated are translated more efficiently than those that are merely capped or polyadenylated (18), although how the simultaneous association of eIF4E and PABP with eIF4G promotes synergistic activation of translation initiation by the 5′ and 3′ ends has not been elucidated in mechanistic terms. A number of reports have however provided useful insight: for example, the binding of wheat PABP to eIF-iso4F enhances eIF-iso4F-cap interactions (19), the binding of yeast Pab1 to RNA increases this protein's affinity for eIF4G (20), while in the same organism the association of eIF4G and of Pab1 with eIF4E stabilizes eIF4E-cap complexation (21–24). Human PABP is thought to stimulate translation initiation by multiple mechanisms, one of which involves enhancement of eIF4G binding to the mRNA (25). Overall, these results convey a picture of mutual enhancement of many of the interactions in the molecular chain mRNA5′-m^7^G-eIF4E-eIF4G-PABP-poly(A)-3′mRNA. At least inasmuch as eIF4E can influence the accessibility of the 5′cap to proteins involved in the catalysis or modulation of decapping, the relationship between the 5′ and 3′ ends of mRNA potentially also plays a role in the control of mRNA degradation (26,27).

Taking as our starting point earlier evidence that N-terminally extended versions of eIF4E (i.e. isomers 3 and 4) can interact with PABP (isomers 2 and 1, respectively; 11,16), this study set out to determine whether an alternative protein-mediated 5′–3′ mRNA interaction chain forms in *Leishmania*, and to characterize key structural features underpinning its assembly. Techniques of NMR, X-ray crystallography, fluorescence anisotropy, microscale thermophoresis and surface plasmon resonance have been used to develop a quantitative understanding of the structures and interactions underpinning this unique type of interaction chain. In particular, we demonstrate the uniquely pivotal structural role of the trypanosomal eIF4E4–PABP1 interaction mediated by the eIF4E4 N-terminal region centered on the PABP-interacting motif 2 (PAM2).

## MATERIALS AND METHODS

### Protein purification

All proteins (and protein complexes) were encoded by synthetic reading frames in the *E. coli* strain NiCo21(DE3; NEB), grown in Terrific Broth medium supplemented with 25 μM ampicillin. Once the culture had reached OD_600_ = 0.6, expression was induced by the addition of 0.5 mM IPTG and then each expression strain was incubated with shaking for 17–22 h at 16°C. The cells were pelleted by centrifugation at 10 000 rpm for 45 min at 4°C in a Beckman-Coulter Avanti J-26 XP centrifuge using a JLA 8.1 rotor, then re-suspended in lysis buffer [40 mM HEPES pH 7.4, 10% glycerol, 0.5 M NaCl, 2 mM DTT, 0.1% Triton X-100, 20 mM imidazole, 1 mM PMSF plus other protease inhibitors (1 tablet of cOmplete™ Mini EDTA-free Protease Inhibitor Cocktail, Roche, per 25 ml of buffer)], using 2 ml of buffer for each gram of cells. The cells were lysed by sonication (4 × 45 seconds on 70% power interspersed with 1min periods on ice). After clarification by centrifugation at 16 000 rpm for 45 min at 4°C in a Beckman–Coulter Allegra 64R centrifuge using F0685 rotor, the supernatant containing soluble proteins was incubated overnight at 4°C with 2 ml of the affinity resin (Amintra Cobalt IDA Resin, Expedeon for His_12_-tagged proteins) that had been equilibrated with the lysis buffer. All affinity purifications were performed in batch mode. After incubation with lysate, the resin was first washed 3 × 20 minutes with 12 ml of lysis buffer (without Triton X-100 and protease inhibitors), and then 3 × 20 min with 12 ml of wash buffer (the same as lysis buffer but without Triton X100, protease inhibitors and with lower salt concentration, i.e. 150 mM NaCl). The proteins were eluted from the resin using elution buffer (wash buffer containing 0.5 M imidazole and 0.1% DDM). Depending on the protein yield, between 4 and 10 elutions were made, each using 2ml of elution buffer incubated for 15min with the resin. Gel filtration was used to separate protein complexes from single proteins and aggregates. Selected elution fractions derived from affinity purification were pooled and concentrated to 0.5ml, using Sartorius Vivaspin 3000 MWCO PES 20 ml concentrators (3000rpm in an Eppendorf 5810R centrifuge). For the highly-produced proteins, and for proteins prone to aggregation, 0.5ml volumes of the best elution fraction(s) were collected for further use. These were loaded onto a Superdex^®^200 Increase 10/300 GL column [GE, separation range 10–600 kDa; flow rate 0.4 ml/min of running buffer (20 mM HEPES, 150 mM NaCl, 2 mM DTT, 5% glycerol)], using a GE AKTA FPLC Purifier UPC100, and 0.6 ml fractions were collected. Fractions were analysed via SDS-PAGE and those containing the purest proteins were selected for further studies. The protein concentrations in the isolated fractions were determined by running at least four different dilutions of the proteins on a stained (InstantBlue™ Coomassie Protein Stain, Expedeon) SDS gel against BSA standards, followed by densitometric analysis using ImageQuant software (GE). The identities of all proteins and protein domains were confirmed using mass spectrometry.

### Mass spectrometry

The identification of proteins was performed via mass spectrometry using nanoLC-ESI–MS/MS. The appropriate protein bands were excised from SDS-PAGE gels and digested with trypsin. The resulting peptides were separated by reversed phase chromatography using an Acclaim PepMap μ-pre-column cartridge 300 μm i.d. × 5 mm 5 μm 100 Å and an Acclaim PepMap RSLC 75 μm × 25 cm 2 μm 100 Å (Thermo Scientific), installed on an Ultimate 3000 RSLCnano system (Dionex). Two mobile phases were used: mobile phase A comprised 0.1% formic acid in water; mobile phase B comprised 0.1% formic acid in acetonitrile. In the next step, the samples were loaded onto the μ-pre-column equilibrated in 2% aqueous acetonitrile containing 0.1% trifluoroacetic acid for 8 min at 10 μl min^−1^ after which peptides were eluted onto the analytical column at 300 nl min^−1^ by increasing the mobile phase B concentration from 4% B to 25% over 22 min then to 90% B over 3 min, followed by a 10min re-equilibration at 4% B. The eluted peptides were then subjected to electrospray ionization and analyzed on a Thermo Orbitrap Fusion system (Q-OT-qIT, Thermo Scientific). Precursor peptides scanning from 375 to 1500 *m/z* were analyzed at 120 K resolution (at 200 *m/z*) with a 2 × 10^5^ ion count target. Tandem MS was performed by isolation at 1.2Th using quadrupole HCD fragmentation with a normalized collision energy of 30 and rapid scan MS analysis in the ion trap. The MS2 ion count target was set to 3 × 10^3^ and the maximum injection time was 200 ms. Precursors with charge state 2–6 were selected and sampled for MS2. The dynamic exclusion duration was set to 30 s with a 10 ppm tolerance around the selected precursor and its isotopes. Mono-isotopic precursor selection was turned on. The instrument was run in top speed mode with a 1 s cycle. Raw MS data were processed using MSConvert in the ProteoWizard Toolkit (version 3.0.5759). The MS2 spectra were searched using the Mascot engine (Matrix Science, version 2.4.1) against the available *Leishmania* database and the common Repository of Adventitious Proteins Database (http://www.thegpm.org/cRAP/index.html). Peptides were generated from tryptic digestion with up to two missed cleavages, carbamidomethylation of cysteines as fixed modifications, and oxidation of methionines as variable modifications. Precursor mass tolerance was 10 ppm and product ions were searched at 0.8 Da tolerances. Scaffold (version Scaffold_4.3.2, Proteome Software Inc.) was used to validate MS/MS-based peptide and protein identifications. Peptide identifications were accepted if they could be established at >95% probability by the Scaffold algorithm and contained at least two identified peptides. Protein probabilities were assigned by the Protein Prophet algorithm (NESVIZHSKII, 2003). Proteins sharing significant peptide evidence were grouped into clusters.

### Peptide synthesis

Two of the peptides (Supplementary Figure S1A,B) were synthesized on a CEM Liberty Blue peptide synthesizer with microwave assistance using default coupling cycles. The synthesis was performed on a 0.1mmol scale using Rink Amide MBHA resin (0.33 mmol/g), DMF as a solvent, 20% piperidine in DMF for the deprotection and DIC and OXYMA pure for couplings. Peptides were N-terminally acetylated or fluorescently labelled with fluorescein carboxylic acid (FAM) attached through a 6-aminohexanoic acid linker. Cleavage from the resin was accomplished using TFA:H_2_O:TIS:EDT, 92.5:2.5:2.5:2.5 (5 ml × 3 h) and peptides were precipitated using cold ether. Pure peptides were obtained after preparative HPLC purification on a Jupiter Proteo 90Å 21.2 × 250 mm reverse phase column using a gradient of 20–60% acetonitrile with 0.1% TFA and lyophilization. High-resolution mass spectrometry (HR-MS) data were recorded using electrospray ionization in positive mode (ESI+) with a Bruker MaXis Impact spectrometer. Analytical HPLC experiments were performed using an Agilent 1290 Infinity LC series system equipped with an Ascentis Express Peptide ES-C18 100 × 2.1 mm column, 2.7 μm particle size on a 5–95% gradient of acetonitrile in water (with 0.1% TFA) over 10 min. The 14mer peptides (labelled with fluorescein isothiocyanate; FITC) used to test the effects of mutations in the PABP1 binding site on eIF4E4 were synthesized and HPLC-purified (to 98% purity) by Thermo Fisher Scientific (Supplementary Figure S1C).

### NMR

Isotopically labelled proteins were prepared by growing the appropriate strains in an Applikon ez- Control Bioreactor. The minimal medium was supplemented with D-glucose (U^13^C6, 99%, Cambridge Isotope Labs) and ammonium chloride (^15^N, 99%). Protein samples were prepared in HEPES buffers (50 mM, pH 7.5) at concentrations in the range 50–200 μM. Experiments were performed on cryoprobe-equipped Bruker 600 and 950 MHz spectrometers in 5mm Shigemi tubes using 300 μl of solution in the presence of 5% D_2_O at a temperature of 300 K. Backbone assignments were accomplished via ^1^H–^15^N-HSQC and the triple resonance experiments HNCA/HNCOCA, HNCACB/HNCOCACB and HNCO/HNCACO. TROSY-based variants were used for experiments involving larger complexes. Data analysis was performed using the software packages Bruker TopSpin 3.5pl7 and CCPN Analysis V2.4.2.

### X-ray crystallography

For crystallization, the protein was concentrated to 20 mg ml^−1^. Crystals of PABP1(J) and PABP1(J) complexed with peptide were grown at 22°C by vapour diffusion in 96-well plates (Swiss-Sci) using a Mosquito liquid handling robot (TTP LabTech) with 100 nl protein mixed with 100 nl well liquor. Crystals of PABP1(J) were harvested from a drop with 0.2 M ammonium sulphate, 0.1 M sodium cacodylate at pH 6.5, 30% PEG8000 (Structure Screen) and those of the protein in complex with the peptide were grown in 0.2 M ammonium sulphate; 0.1 M MES; 20% PEG 8000 (ProPlex). Both were cryo-cooled by plunging the crystals directly into liquid nitrogen. Data were collected at Diamond Light Source beamlines IO3 (PABP1(J)) and I24 (PABP1(J) + peptide) and processed at the beamline with XDS through the Xia2 pipeline. Further processing was carried out in CCP4. The structure of PABP1(J) was solved by molecular replacement in Phaser using the structure of an MLLE domain from human PABP1 (PDB accession 3PTH) as a search model. Refinement was carried out using the PHENIX package. The model was first rebuilt using PHENIX.autobuild and thereafter refined using a combination of Phenix.refine and manual intervention using Coot. The structure of the complex with peptide was also solved by molecular replacement using the refined structure of the PABP1(J) domain alone as the search model. The structure was refined as above. Individual chains were defined as TLS groups in the refinement of both structures. Interactions between amino acid residues in the crystal structure were displayed using EMBL/EBI software LIGPLOT+ (version 4.5.3; https://www.ebi.ac.uk/thornton-srv/software/LIGPLOT). Both structures contain two PABP1(J) domains in the asymmetric unit, which are covalently linked through a disulphide bridge between cysteine 552 of each molecule. Data collection and refinement statistics are shown in Supplementary Tables S1 and S2. The Worldwide Protein Data Bank access codes for the structure depositions are 6H7A [PABP1(J)] and 6H7B [PABP1(J) + eIF4E4 peptide]. Full references for the methods used are given in the Supplementary Data section.

### Electrostatic surface modelling and hydrophobicity surface plots

Electrostatic surface modelling was performed using the PyMOL V2.0.5 – APBS (Adaptive Poisson–Boltzmann Solver) plugin (28,29). Hydrophobicity surface plots were generated using PyMOL script Color h, http://us.expasy.org/tools/pscale/Hphob.Eisenberg.html (30).

### MST, SPR, fluorescence anisotropy measurements

Microscale thermophoresis experiments were performed using eIF4E4(iv) or eIF4G3 labelled with a fluorescent tag (Monolith Protein Labeling Kit Red-Maleimide), or the eIF4G3 peptide VEQIRSVRNNYLEPPYPGFSLDEVVR labeled with fluorescein-5-isothiocyanate. The concentration of the fluorescently labelled protein (eIF4E4(iv)) was held constant, while the concentration of non-labelled ligand protein was varied. The experiment was conducted by preparing 16 tubes with various ratios of the interacting labelled/non-labelled proteins. The solutions were introduced into capillaries, which were then loaded into a Nanotemper Monolith NT.115 instrument for measurement. For the SPR experiments, eIF4E4(iv) was immobilized via amine coupling to a Biacore CM5 sensor chip (GE Healthcare Life Sciences) and titrations against PABP1(J) were performed in a Biacore T200 instrument. In order to maximise confidence in the calculated dissociation constant values (based on *k*_ON_ and *k*_OFF_ values), two types of model were fitted to the raw data: a homogeneous 1:1 binding model, and a heterogeneous 1:1 two-ligand model. The heterogeneous model was found to give better fits. This is not unexpected, since the mode of protein coupling used tends to result in a population of protein molecules that are heterogeneously oriented with respect to the SPR chip surface (see Supplementary Figure S7). Fluorescence anisotropy measurements were performed using a PTI QuantaMaster fluorimeter. To calculate the *K*_D_, normalized anisotropy was plotted versus the concentration of added eIF4E4(v)::PABP1(J), and the data were fitted using the general equation y = a-b*c∧x with the help of the OriginLab model Asymptotic 1.

## RESULTS

### Identification of the PABP1–eIF4E4 interaction domains

**Figure 1.**
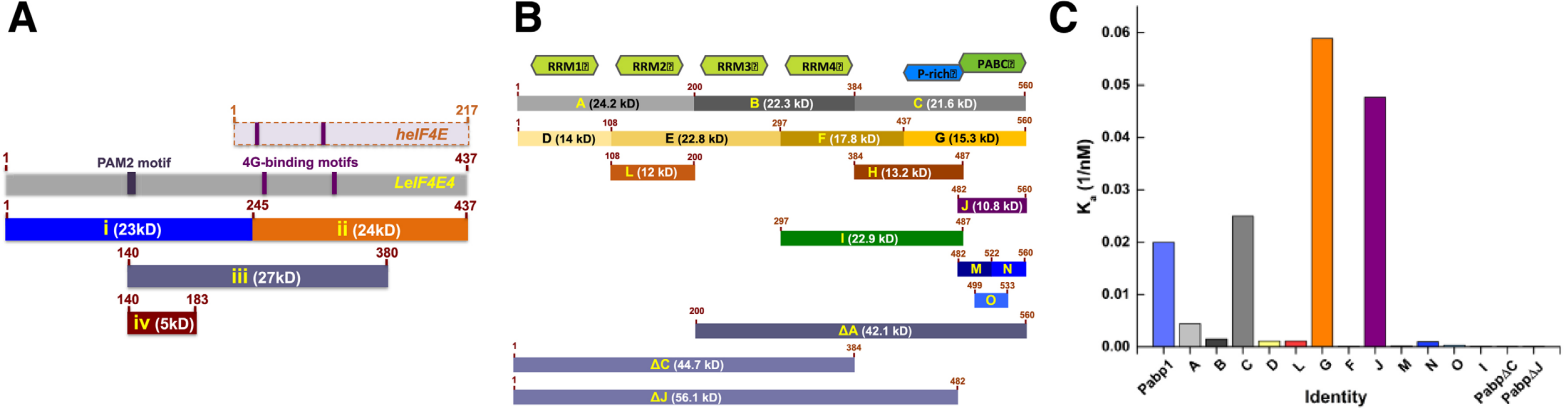
Screening regions of *Leishmania major* eIF4E4 and PABP1 for binding. (**A**) Different parts of the eIF4E4 reading frame were subcloned and expressed in *E. coli*. The overall structure of human eIF4E is shown for comparison. (**B**) Similarly, different sections of *Leishmania* PABP1 reading frame were expressed in *E. coli*, purified and screened for binding to eIF4E4(iv) using microscale thermophoresis (Supplementary Figure S3). The estimated affinities (*K*_a_ values) between eIF4E4(iv) and the respective PABP1 sections are summarized in panel (**C**).

Our first objective was to characterize the interactive properties of eIF4E4 and PABP1 from *Leishmania* and to define the domains from the respective proteins that mediate these interactions. We approached this by preparing *Escherichia coli* expression constructs, as described in Figure 1A, B, that cover all potential interaction sites. The full-length eIF4E4 and PABP1 DNA reading frames were generated synthetically in codon-optimised form for expression in *E. coli*. A polyhistidine affinity tag (His_12_) was introduced at the N-terminal or C-terminal end of each protein domain used in this study (Supplementary Figure S2), thus providing tagged derivatives that could be employed in different combinations with untagged proteins in the various biophysical procedures used for quantitative characterization. By studying the behavior of the respective recombinant protein domains indicated in Figure 1A,B, we discovered that a number of them, especially the larger ones, were prone to aggregation. Fortunately, by creating a large number of (overlapping) domain constructs we could identify a subset of protein domains with suitable solubility characteristics that covered all sections of PABP1 and the extended N-terminal region of eIF4E4. The selected protein domains were then analyzed in microscale thermophoresis binding assays (Figure 1C; Supplementary Figure S3), leading to a categorization of the domains into groups that manifested strong binding and other groups that engaged in relatively weak binding. The main outcome of these experiments was identification of a strong interaction between the PABC domain of PABP1 (here prepared as segment J; Figure 1B) and an approximately 5 kDa region [segment (iv); Figure 1A] of the extended N-terminal domain of eIF4E4. Analysis of the sequence of eIF4E4(iv) revealed the presence of a candidate PAM2 motif, a consensus recognition site found in other proteins that bind to poly(A)-binding proteins (31,32). This finding is consistent with the results of previous (qualitative) pull-down and two-hybrid assay experiments performed with PABP1 and eIF4E4 (7). Another report has attempted to predict molecular interactions between *Leishmania* PABP1 and eIF4E4 by modeling MLLE and PAM2 domains from other (human) proteins (33). In order to obtain definitive experimental information on the interaction architecture of the *Leishmania* proteins, we decided to perform structural analysis on the PABP1 and eIF4E4 domains identified by our interaction screening procedure.

### Structural analysis of PABP1–eIF4E4 interactions

**Figure 2.**
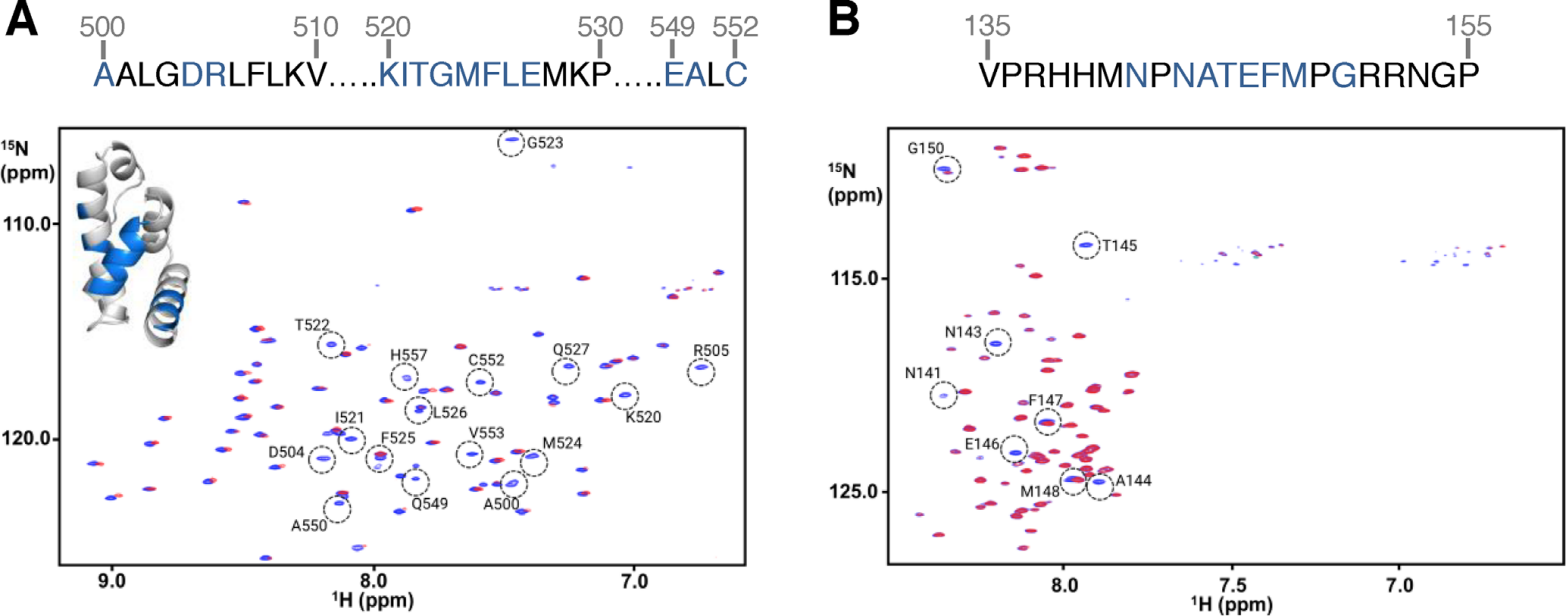
NMR spectra reveal interacting residues and conformational changes. (**A**) ^1^H–^15^N HSQC spectra of ^15^N,^13^C-labelled PABP1(J) in the absence (blue) and presence (red) of unlabeled eIF4E4(iv), with residues undergoing chemical shift perturbations (CSPs) upon binding highlighted. (**B**) The equivalent experiments were performed using ^15^N–^13^C-eIF4E4(iv) plus unlabelled PABP1(J). The residues affected by binding are also highlighted in the sequence for PABP1 (blue highlighted amino acids at the top of panel **A**) and in the sequence for eIF4E4 (blue highlighted residues at the top of panel **B**).

We prepared uniformly ^15^N-,^13^C- labeled PABP1(J) and eIF4E4(iv) and used these protein domains, together with the non-labelled versions, for NMR analysis. By this means, we were able to identify the amino acids comprising the respective binding motifs (Figure 2). The ^1^H–^15^N-HSQC NMR spectra showed PABP1(J) to be folded (Figure 2A), while eIF4E4(iv) displayed amide resonance chemical shift dispersion characteristic of an intrinsically disordered domain (Figure 2B). Addition of either unlabeled domain to the other in uniformly ^15^N–^13^C-labelled form induced selective chemical shift perturbations indicative of specific binding. Binding of unlabeled eIF4E4(iv) to ^15^N-^13^C- labeled PABP1(J) (Figure 2A) enabled us to identify interactions involving residues in PABP1 that manifest homology to the 70-residue MLLE (here M**F**LE) domain (34). In the reciprocal experiment, addition of PABP1(J) to ^15^N–^13^C-eIF4E4(iv) caused major chemical shift perturbations (CSPs) that map to the region N141-G150 of eIF4E4 (Figure 2B), which matches the putative PAM2 motif that we identified via our initial binding studies (see above). In an additional experiment, we assessed the impact of adding a short (14-amino-acid-long) unlabelled synthetic peptide centred around the eIF4E4 PAM2 motif to ^15^N–^13^C- labeled PABP1(J) (Supplementary Figure S4A). The resulting CSPs in the PABP1(J) ^1^H–^15^N-HSQC spectrum mapped exactly onto those observed in Figure 2A, thus confirming the major role of the PAM2 motif in determining the specific association of eIF4E4 with PABP1.

**Figure 3.**
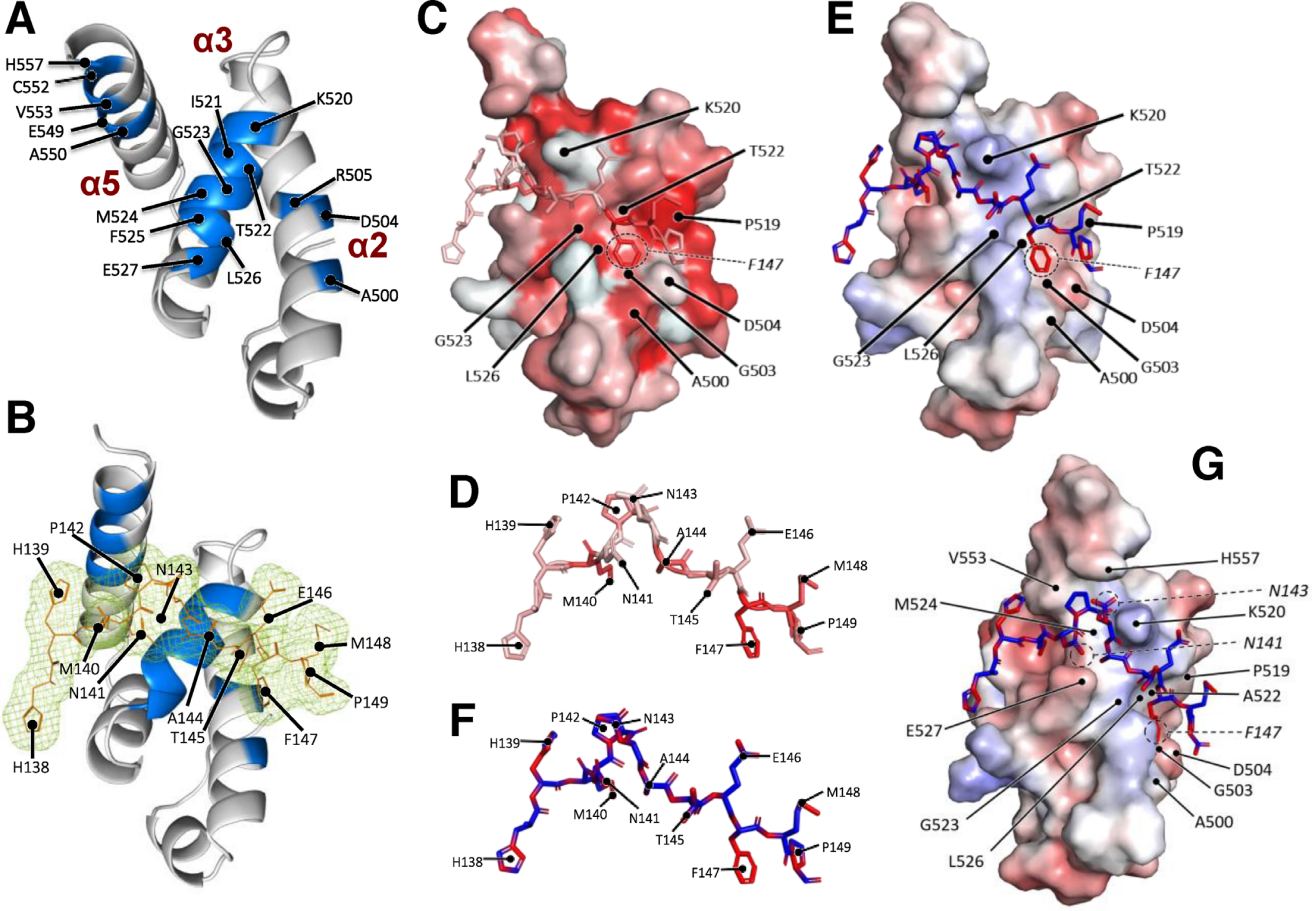
X-ray crystallographic analysis of the *Leishmania* PABP1 PABC domain binding to eIF4E4. (**A**) Crystal structure of PABP1(J), featuring the α-helices and location of the MFLE motif (residues 524–527) in the PABC domain. (**B**) Electron density of the eIF4E4 PAM2 peptide overlaid over the PABP1 PABC1 structure. (**C**) Hydrophobicity surface map of the PABP1 PABC structure together with the bound backbone structure of the eIF4E4 PAM2 peptide. The intensity of the red shading increases with predicted hydrophobicity. (**D**) Enlarged and fully annotated backbone structure of the eIF4E4 PAM2 peptide showing predicted hydrophobicity features. (**E**) Electrostatic potential surface map [highlighting relatively more positively charged (basic; blue) and relatively more negatively charged (acidic; red) regions] of the PABP1 PABC structure overlaid with the backbone structure of the eIF4E4 PAM2 peptide. (**F**) Enlarged and fully annotated eIF4E4 peptide backbone structure colour-coded according to electrostatic potential. (**G**) Image in panel **E** rotated to improve view of interactions with the N-terminal half of the eIF4E4 peptide. More details are provided in Supplementary Figure S5.

In further work, we were able to solve the crystal structures of PABP1(J) (Figure 3A) and of PABP1(J) co-crystallized with a synthetic eIF4E4 PAM2 peptide (Figure 3B), both to a resolution of ∼2 Å (Supplementary Tables S1 and S2). Obtaining these structures enabled us to locate the binding motif for eIF4E4 in relation to the three-dimensional structure of *Leishmania* PABP1 (Figure 3B, C, E, G). The structure of the complex reveals how the eIF4E4 peptide binds across the three α-helical segments of the 70-residue PABC domain of PABP1 in a similar fashion to other PABC-interacting proteins (34). The peptide (Figure 3D, F) includes the PAM2 motif (**M**N**P**N**A**TE**F**M**P**) of eIF4E4, which follows the same pattern of PAM2 motifs found in other proteins, including Paip1 and eRF3 [Φ-(P/V)-A—F-P (34)]. The eIF4E4 peptide in the crystal structure assumes an extended conformation, whereby the PAM2 motif wraps around the core KITGMFLE motif of the PABC domain of PABP1 (Figure 3B, C, E, G). Consistent with the structures of other analogous complexes, binding is associated with relatively minor changes in the conformation of the PABP1 fragment; the side chains of only Lys 520 and Glu 527 are affected.

The phenylalanine residue in the PAM2 motif (here F147, circled in Figure 3C, E) fits into a large pocket formed of multiple residues on the PABC domain, including three of the KITGMFLE motif residues (Supplementary Figure S5). The proline at position 142 (Figure 3D, F) reorients the path of the eIF4E4 peptide across the three helices, thus influencing the configuration of the multiple interactions that can be identified between the two partner molecules in the complex (as highlighted in the LIGPLOT representation in Supplementary Figure S5E). Next to this proline is an asparagine (N143; Figure 3F, G) whose polar and aliphatic side chain would not be expected to engage in significant interactions with the PABP1 protein (Figure 3A, B, G). The phenylalanine (F525) in the PABC core motif of *Leishmania* PABP1 (KITGM**F**LE) represents a variation on the MLLE motif generally found in other poly(A)-binding proteins; this residue is located on α-helix 3 and forms part of a hydrophobic pocket that interacts with M140 in the eIF4E4 sequence (Figure 3C,D; Supplementary Figure S5A,E). A144 in eIF4E4 fits into the hydrophobic pocket formed partially by the PABP1 G523 that also sits on α-helix 3 (Figure 3A, C, D). The carbonyl oxygens of both P142 and A144 interact with the Nϵ atom of K520 at the N-terminal end of α-helix 3 (Figure 3E, F). It is also noteworthy that the crystal structure indicates that M148 is angled away from (and does not interact with) P519 (Figure 3G; Supplementary Figure S5C,E), although our NMR data (Figure 2B) reveal that the M148 backbone amide undergoes a significant change in its environment upon binding to PABP1. Overall, the multiple interactions identified here (most of which are highlighted in the LIGPLOT in Supplementary Figure S5E) provide a basis for understanding the molecular principles underpinning tight binding between eIF4E4 and PABP1.

### Mutational analysis of the roles of conserved residues in eIF4E4

The structural data prompted us to use mutational analysis to explore further the role of conserved residues in the eIF4E4 PAM2 motif in binding. In order to do this, we performed microscale thermophoresis experiments using a set of FITC-labelled synthetic 14mer peptides (Table 1; Supplementary Figure S1C) in binding experiments with PABP1(J). Considering first the mutations affecting hydrophobic residues, we found that single-site alanine substitution of the methionines at positions 140 and 148 had minimal impacts on binding affinity [compared to the affinity calculated for the ‘wild-type’ peptide (Table 1)]. The M140A result indicates that the side chain of alanine (which, like that of methionine, is categorized as hydrophobic) can substitute adequately for that of methionine as a moiety that can interact with the hydrophobic pocket partly formed by F525 in PABP1, whereby other compensating interactions could also potentially be involved. The small effect on binding affinity of M148A, on the other hand, is consistent with the observation that this residue is oriented away from the PABP1 protein surface (Figure 3G; Supplementary Figure S5C). Moving on to position 147, we see that an alanine does not achieve as energetically favourable a fit into the hydrophobic pocket on PABP1 (Supplementary Figure S5) as F147, since the F147A mutant peptide manifests a markedly reduced affinity relative to the wild-type sequence (Table 1).

**Table 1.** PAM2-motif FITC-labelled peptides

Mutations	Sequence	*K*_D_ (nM)
WT	HH**M**NP**N**AT**EFM**PGR	33.0 ± 0.9
M140A	HH**A**NPNATEFMPGR	34.3 ± 1.3
N143A	HHMNP**A**ATEFMPGR	69.2 ± 4.9
E146A	HHMNPNAT**A**FMPGR	163.0 ± 16.4
F147A	HHMNPNATE**A**MPGR	127.3 ± 13.4
M148A	HHMNPNATEF**A**PGR	62.8 ± 3.1
5 x A	HH**A**NPA**A**T**AAAP**GR	n.d.*

*not detectable

We next looked at the effects of substituting amino acids with charged or polar side chains. Replacement of the glutamic acid residue at position 146 by alanine also leads to a strongly reduced affinity value in this assay; this is likely to be due to the loss of the predicted electrostatic interaction between eIF4E4 E146 and PABP1 K520 (Figure 3E). In contrast, N143A had no effect on binding (Table 1), a result that is consistent with the structure-based prediction that N143 does not engage in strong interactions with PABP1. Finally, the effect on binding of simultaneous multiple alanine-substitutions of all of the individual PAM2 motif residues described above confirms their necessity for detectable binding (Table 1; peptide 5 x A), and also emphasizes the importance of a combination of different atomic interactions.

### Characterization of a PABP1–eIF4E4–eIF4G3 complex

**Figure 4.**
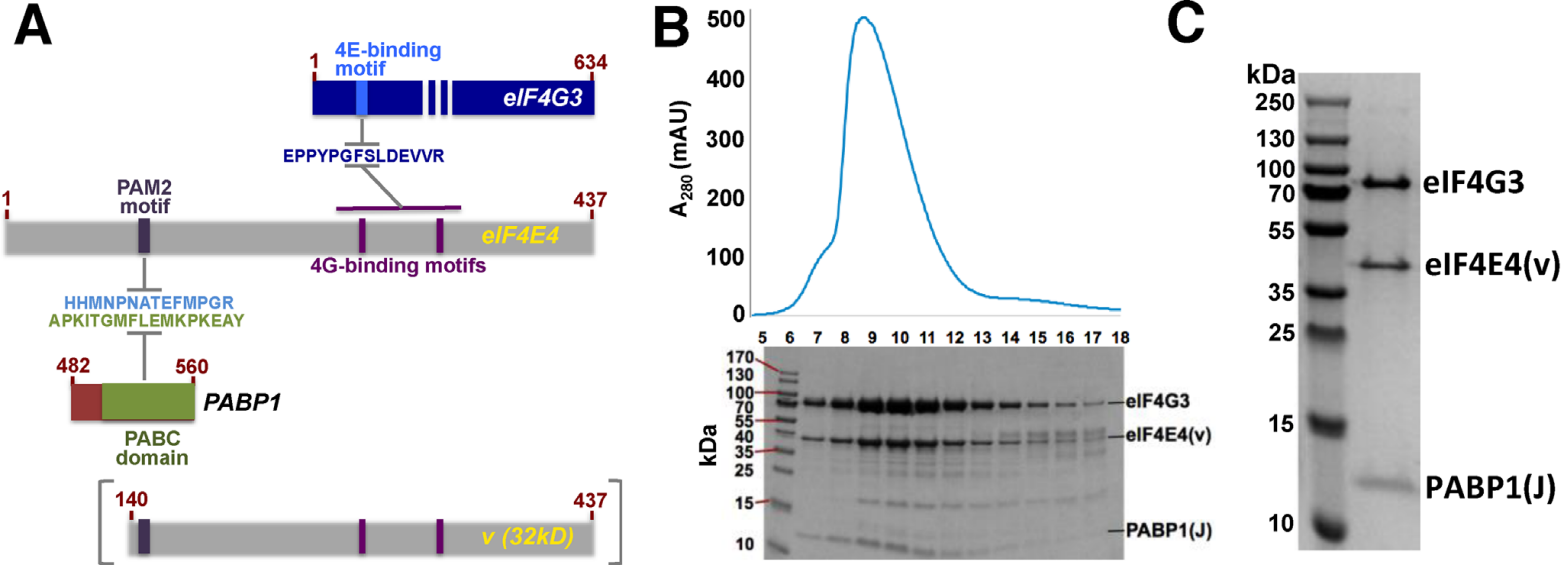
Formation of a PABP1:eIF4E4:eIF4G3 complex. (**A**) Overview of proteins and protein segments co-synthesized in *E. coli*, featuring PABP1(J) and eIF4E4(v) as well as relevant amino acid sequence motifs. (**B**) Co-migration of eIF4G3-His_12_, eIF4E4(v) and PABP1(J) proteins on a Sephadex 75 gel filtration column after elution from a cobalt affinity column. (**C**) Collection of fractions 7–8 yielded a highly pure preparation of the complex: PABP1(J)-eIF4E4(v)-eIF4G3. The identities of these proteins were confirmed using mass spectrometry.

Given that the eIF4E4 factor carries both the PABP1-binding (PAM2) site in its N-terminal extension (Figure 1A) and eIF4G3-binding-motifs on its dorsal face, we next examined whether a longer *Leishmania* eIF4E4 fragment (segment v; Figure 4A) could serve as the platform for formation of both heterodimeric and heterotrimeric complexes as the result of co-expression *in vivo*. Taking into account our previous observations of the propensity of some of the synthetic *Leishmania* protein domains to aggregate at high concentrations, we used a dual expression plasmid to support synthesis of PABP1(J) in parallel with eIF4E4(v) (Supplementary Figure S2). Co-expression was successful, in that it not only led to incorporation of almost all of the synthesized eIF4E4(v) protein into the expected PABP1(J):eIF4E4(v) complex, but thereby also suppressed much of the aggregation and degradation that was otherwise observed when eIF4E4(v) was synthesized alone in *E. coli*.

Development of an improved procedure to isolate a stable and soluble heterodimeric complex encouraged us to attempt formation of a heterotrimeric complex that would throw more light onto the eIF4E4-mediated interactions that bridge the 5′ and 3′ ends of mRNAs in *Leishmania*. Since eIF4E4(v) included the putative binding motifs for both PABP1 and eIF4G3 (Figure 4A), we tested the hypothesis that eIF4E4(v) would be able to form a scaffold upon which the two other protein domains could be assembled. We showed that this heterotrimeric complex could indeed be formed via two types of experiment. First, we used cobalt-column affinity chromatography followed by gel filtration chromatography to identify complexes formed upon mixing eIF4E4(v), PABP1(J) and full-length eIF4G3 (Figure 4B). By this means, we were able to isolate a soluble heterotrimeric eIF4E4(v):PABP1(J):eIF4G3 complex that, in the initial purification step, had been selected on the basis of its association with eIF4G3-His_12_ (Figure 4C).

In the second type of experiment, we collected ^1^H-^15^N- HSQC spectra of the heterodimeric complex formed by ^15^N-,^13^C-labelled PABP1(J) and ^15^N-,^13^C- labeled eIF4E4(v), and found that the addition of a synthetic 28mer peptide that includes the eIF4G3 motif (35) that binds the eIF4E4 dorsal face induced specific NMR CSPs (Supplementary Figure S4B). Since the PABP1(J) domain contains only the PAM2-binding PABC site, these data suggest that eIF4G3 can participate in a heterotrimeric eIF4E4:PABP1:eIF4G3 complex by virtue of an interaction with eIF4E4. In combination with fluorescence anisotropy data described in the next section, this indicates that, like eIF4E proteins from other organisms, *Leishmania* eIF4E4 has a dorsal binding domain structure for binding to the N-terminal region of eIF4G3 that can support heterotrimeric complex formation (Figure 4; Supplementary Figure S6). The eIF4E4 residues thought to be directly involved in eIF4G3 binding differ from those involved in complexation of the human eIF4G and eIF4E proteins (7). However, the functional role of these dorsal binding interactions is equivalent to that found in other eIF4E species, and we therefore did not prioritize further characterization of these CSPs in the present study. Instead, we next focused on the question as to whether we could detect any form of interaction between eIF4G3 and PABP1. Most eukaryotic eIF4G proteins possess an N-terminal PABP-binding site that binds to the RRM1–2 domain of PABP (2). Although sequence comparisons reveal that the *Leishmania* eIF4G proteins lack the directly equivalent N-terminal region (Supplementary Figure S6A) we could not, on this basis alone, exclude the possibility that a different type of site with a corresponding role might exist in an alternative domain context.

### Unique interaction architecture underpinning the trypanosomal 5′–3′ bridging complex

**Figure 5.**
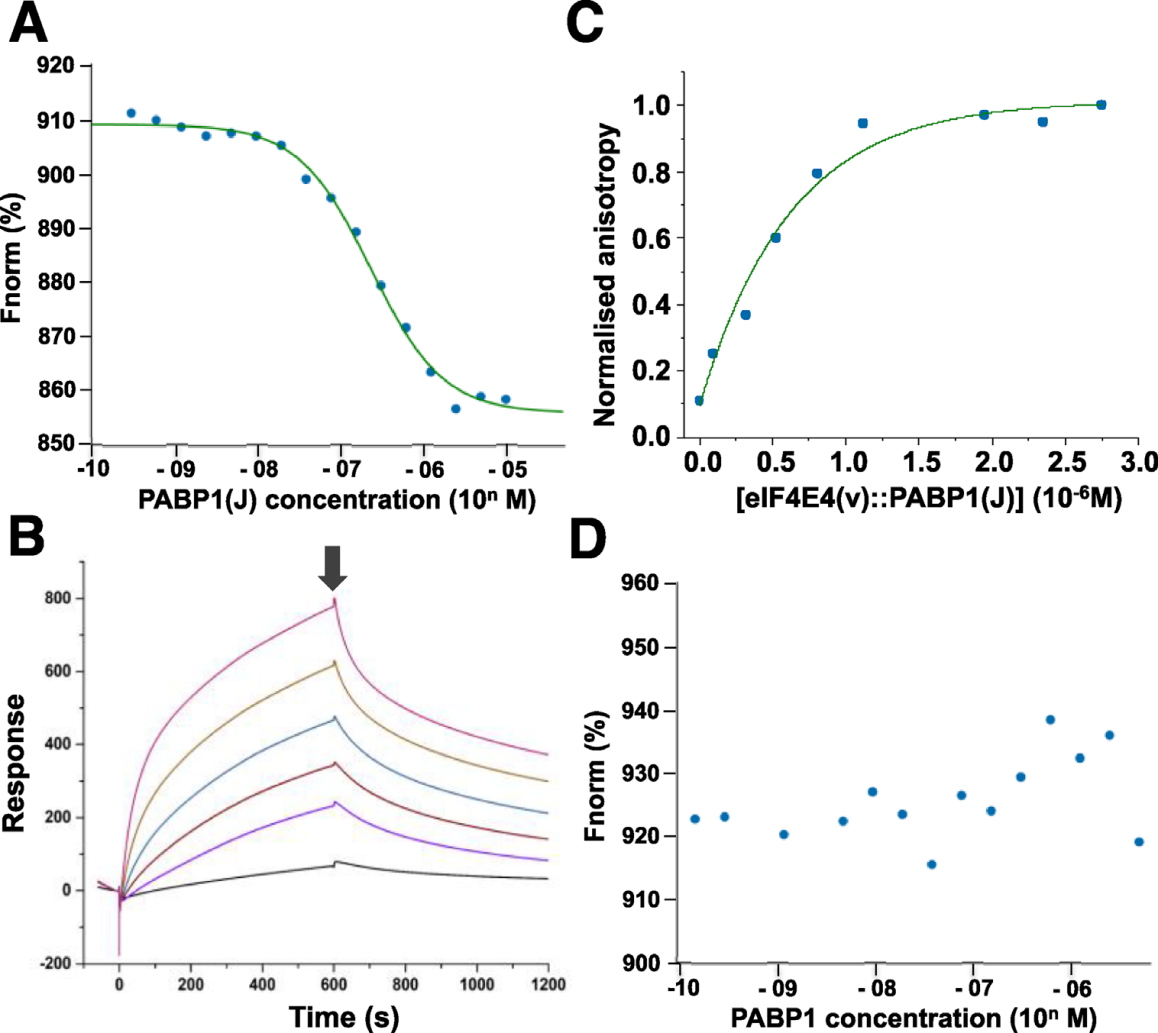
Quantitative characterization of interactions of *Leishmania* cap-complex proteins. Microscale thermophoresis titrations (**A**) of Alexa-647-labelled eIF4E(iv) against non-tagged PABP1(J) domain and surface plasmon resonance (SPR) experiments (**B**) confirmed tight binding between eIF4E4(iv) and PABP1(J) (compare Figure 3). In the SPR measurements, eIF4E4(iv) was immobilized on the chip surface and PABP1(J) was passed over the chip at concentrations of 0.02 (black line), 0.09, 0.19, 0.37, 0.74 and 1.49 (pink line) μM (binding phase). At the time-point indicated by the arrow, the protein solution was replaced by buffer only, thus initiating the release phase. The best fits to the SPR data were obtained using using a heterogeneous binding model (Supplementary Figure S7; Supplementary Table S3). Fluorescence anisotropy measurements (**C**) detected binding of Alexa-647-labelled eIF4G3 to the complex eIF4E4(v):PABP1(J). In contrast, no binding could be detected between Alexa-647-labelled eIF4G3 and PABP1 (**D**). The respective binding data are summarized in Supplementary Table S3.

We wanted to establish a quantitative understanding of the relative significance of interactions between the *Leishmania* cap-binding complex factors eIF4E4, eIF4G3 and PABP1 using protein domains or full-length proteins, rather than peptides. We selected analytical methods that can be applied at low protein concentrations, thus minimizing the occurrence of aggregation-related artefacts: microscale thermophoresis, surface plasmon resonance and fluorescence anisotropy (Figure 5; Supplementary Table S3). Microscale thermophoresis was performed using proteins that had been labelled using Alexa 647. We observed tight binding between eIF4E4(iv) and PABP1(J) (*K*_D_ = 0.22 × 10^−7^ M; Figure 5A). High-affinity binding between eIF4E4(iv) (in this case immobilized) and PABP1(J) was confirmed by surface plasmon resonance measurements. In this case, a heterogeneous model assuming a combination of two different binding states of eIF4E4(iv) generated good fits to the data (the average of the calculated *K*_D_ values for the two states in this model (*K*_Dav_) = 3.3 × 10^−7^ M; Supplementary Figure S7, Supplementary Table S3). Given the predicted role of PABP1 F525 in forming the hydrophobic pocket that the eIF4E4 M140 side chain fits into, and the fact that it is unusual to find a phenylalanine substituted for a leucine in the MLLE motif, we also tested the importance of F525 in the interaction with eIF4E4. Mutation of this phenylalanine to an alanine [PABP1(J) F525A] resulted in a major loss in binding affinity (*K*_D_ = 5.5 × 10^−7^ M; Supplementary Table S3) as measured using microscale thermophoresis. The PABP1(J) F525A *K*_D_ value is 25 times greater than that of the corresponding non-mutated PABP1(J) value.

Fluorescence anisotropy experiments revealed that a high-affinity interaction (average of calculated *K*_D_ values = 3.4 × 10^−7^M) also occurs between Alexa-647-labelled eIF4G3 and the complex eIF4E4(v):PABP1 (Figure 5C). The latter binding result was confirmed through microscale thermophoresis experiments using full-length eIF4G3, although in this case partial fluorescence quenching limited the accuracy of the *K*_D_ calculation and this result qualifies only as supporting evidence (Supplementary Figure S8A). In contrast, microscale thermophoresis detected no binding between eIF4G3 and PABP1 (Figure 5D; see also control in Supplementary Figure S8A), and this negative outcome was also reflected in pull-down results obtained with lysates from *E. coli* strains that co-produce these two proteins (Supplementary Figure S8B). Finally, microscale thermophoresis analysis of the binding between full-length PABP1 and eIF4E4(iv) revealed no effect of the presence of poly(A) ribonucleotides of length 12 or 20 on the affinity between these two proteins (data not shown).

## DISCUSSION

**Figure 6.**
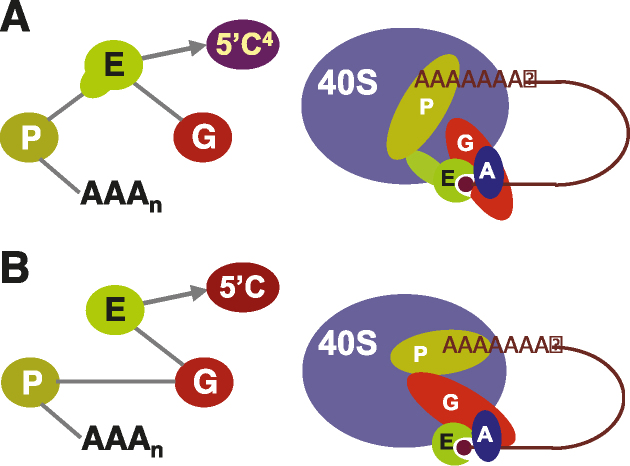
The ‘scaffolding’ component in the trypanosomatid cap-binding complex is eIF4E4. (**A**) The structural data and quantitative interaction measurements reported here demonstrate that *Leishmania* eIF4E4 (E) engages in specific interactions (continuous edges in the interaction graph) with PABP1 (P) and with eIF4G3 (G), whereas there was no detectable interaction between PABP1 and eIF4G3. The known interactions between PABP1 and poly(A), and between eIF4E4 and the cap4 (5′C^4^) structure, complete the map of the eIF4G3–eIF4E4(cap4)-PABP1-poly(A) biomolecular chain. (**B**) In all other known eukaryotic cap-binding complexes, the core scaffolding protein is eIF4G, as reflected in this interaction map.

In this study, we have demonstrated that the N-terminal extension found in *Leishmania* eIF4E4 acts as a focal structural element in formation of a unique type of eukaryotic cap-binding complex architecture. Indeed, because the N-terminal extension adds a PABP1 binding site to the cap- and eIF4G- binding sites that are generally present in previously described eIF4E species from across the kingdoms, the *Leishmania* eIF4E4 factor replaces eIF4G as the core ‘scaffolding’ protein in the cap-binding complex (Figure 6A). We conclude that this N-terminally extended eIF4E anchors eIF4G and PABP1 directly to 5′-end-cap4-modified mRNAs.

While the ‘scaffolding’ function in cap-complex assembly described here is an exceptional role for an eIF4E protein, it also serves to underline the wider importance of some form of molecular bridging between the cap-binding complex and the poly(A) binding protein in eukaryotic cells. In many other organisms, strong binding between eIF4G and PABP promotes translation (17,20,23,25). We find that participation in this core bridging function is not evident in the *Leishmania* eIF4G isomer that binds to eIF4E4. We have used two independent quantitative measurement techniques to resolve uncertainty created by contradictory results from previous attempts to detect binding between eIF4G3 and PABP1 that were based on qualitative pull-down experiments and two-hybrid assays (16,36,37). Our conclusion is that these two proteins do not participate in a readily detectable interaction, and that if there is any (potentially non-specific) binding between the two, this is at a much lower affinity than that measured for the pairs eIF4E4–PABP1 and eIF4E4–eIF4G3.

The N-terminally extended versions of eIF4E (isomers 3 and 4) have been described as essential proteins that are present in at least the promastigote stage of the trypanosomatid developmental cycle (7). These two isomers are of a higher abundance than the other trypanosomatid eIF4E isomers and are the only two for which there is evidence of involvement in cap-complexes implicated in general translation, whereby more recent reports have emphasized the primacy of eIF4E4 in promoting cap-dependent initiation (7,37). In this context, it is noteworthy that the *Leishmania* eIF4E3 N-terminal region possesses a sequence that is only partly related to the PAM2 motif found in eIF4E4 (Supplementary Figure S6B). Moreover, there is considerably less similarity in the PABC domain (outside of the KITGMLLE motif) between PABP1 and the PABP isomers 2 and 3 within *L.major* than there is similarity between the equivalent region of PABP1 sequences across nine species of *Leishmania* and *Trypanosoma* (Supplementary Figure S6C,D). Overall, these observations suggest that eIF4E4-mediated mRNA 5′–3′ bridging interactions play a distinct and possibly dominant role in *Leishmania* translation initiation. In addition, these sequence alignment results alert us to the possibility that a comparable emphasis on the eIF4E4–PABP1 interaction may apply in other *Leishmania* (and *Trypanosoma*) species (Supplementary Figure S6D). We conclude that the role of eIF4E3 requires clarification. Like eIF4E4, it is exclusively present in the cytoplasm of promastigotes, but its mRNA cap binding affinity is lower than that of eIF4E4 (7) and, as we have seen, there are significant differences between the key motifs in the N-terminal extensions of the respective isomers (Supplementary Figure S6B). Future work will need to determine whether eIF4E3 participates in the assembly of a complex (involving eIF4G4 and PABP2) of equivalent structural and functional importance to that of eIF4G3:eIF4E4:PABP1.

From the X-ray and NMR structural data in this study we have learned that there are a large number of atomic interactions underpinning binding between eIF4E4 and PABP1. By performing microscale thermophoresis binding assays using FITC-14mer peptides carrying sequence variants, we have deepened understanding of the relative significance of the respective amino acids around the PAM2 motif in eIF4E4. These experiments have demonstrated the significant roles of E146 and F147, and the at most minor roles of N143 and of M148, in binding. In the case of N143, the NMR data indicated that binding between eIF4E4 and PABP1 has an effect on the environment of this residue, but the X-ray results indicate that it does not engage in significant interactions. In addition, the peptide binding results (Table 1) demonstrate that a combination of multiple interactions, including the partitioning of multiple (hydrophobic) amino acid side chains into non-aqueous regions (pockets) shared by the two proteins (Supplementary Figure S5), underpins binding. This combination of polar, electrostatic and hydrophobic interactions shows some similarity to the molecular basis of binding observed in the protein pairs PABPC1-Paip2 and EDD-Paip1 (34), particularly in terms of engagement of the PAM2 motif (eIF4E4) with hydrophobic pockets on either side of α-helix 3 (on PABP1). Part of the region of *Leishmania* PABP1 that eIF4E4 interacts with is the MFLE motif, which is a variant of the standard MLLE motif, and using a mutated PBAP1(J) protein domain we have confirmed the importance of F525 as a key part of the hydrophobic pocket that accommodates M140 of eIF4E4.

Our NMR analyses have also revealed that the N-terminal extension of eIF4E4 is naturally disordered, and that interaction with PABP1 induces at least partial folding of this region. Unfolded-folded transitions have been shown to be a common theme in other binding processes involving cap-complex factors; for example as the result of interactions between human eIF4E and eIF4G (38), and between human eIF4E and 4E-binding proteins (39). There are also many other instances in nature in which unfolded-folded transitions are thought to be triggered by protein-protein interactions (40). Moreover, PAM2 motifs generally occur in naturally disordered regions (41), as is the case in *Leishmania* eIF4E4. Since there is evidence that phosphorylation of disordered PAM2-containing protein regions can modulate binding to PABC domains (42), the observation that the N-terminal region of eIF4E4 is the target of multiple phosphorylation events (11) suggests that this type of modification might regulate the molecular interactions of eIF4E4.

Previous studies of initiation complexes in yeast, mammalian and plant systems have revealed evidence of cooperativity of the interactions between components in the molecular chain mRNA5′-m^7^G-eIF4E-eIF4G-PABP-poly(A)-3′mRNA (19–25). Of particular relevance here are published data obtained with yeast and human proteins indicating modulation of PABP-eIF4G interactions by poly(A) binding to PABP (20,43). As we have seen, the *Leishmania* eIF4G3–eIF4E4(cap4)-PABP1-poly(A) biomolecular chain lacks direct binding between eIF4G3 and PABP1, and this raised the question whether the binding of poly(A) ribonucleotides to PABP1 might instead modulate the binding affinity between PABP1 and eIF4E4. Our results suggest that the changes in the roles of the respective translation factors in *Leishmania* have created a biomolecular chain that functions without poly(A)-mediated modulation of protein-protein interactions. This outcome is consistent with the observation that the PABP1 PABC domain forms a stable fold in solution that is only minimally altered by binding of the eIF4E4 peptide.

It is also noteworthy that, although still classifiable as of relatively high affinity, the interactions between the *Leishmania* cap-binding-complex proteins reported here are weaker than those reported previously for eukaryotic eIF4E and eIF4G (for example in yeast the *K*_D_ for this interaction = 2.3 × 10^−9^ M; 44). The apparently lower stability of the *Leishmania* interactions could potentially signify that the translation initiation complexes in at least the promastigote form of this organism are more dynamically assembled and disassembled than in other eukaryotes, but more work will be required to test this hypothesis. It is also of interest that the dissociation constant value estimated using microscale thermophoresis for PABP1(J) binding to the eIF4E4(iv) protein domain (*K*_D_ = 0.22 × 10^−7^M) is very similar to that observed for PABP1(J) binding to the 14mer eIF4E4 peptide (K_D_ = 0.33 × 10^−7^ M). This suggests that the primary determinants of eIF4E4 binding are contained in the PAM2 motif.

Finally, this quantitative study has focused on key interactions and structures underpinning the unique architecture of the *Leishmania* eIF4G3–eIF4E4(cap4)–PABP1–poly(A) biomolecular chain that links the 5′ and 3′ ends of trypanosomatid mRNAs. We anticipate that future work will build on these insights to help expand this picture by exploring a wider network of interactions involving other translation factors, such as the DEAD-box RNA-binding protein eIF4A. We note that such future work could reveal further interaction interfaces that may be worthy of consideration as potential drug targets.

## Supplementary Material

Supplementary Data
