# Supplementary material for: The Leishmania PABP1–eIF4E4 interface: a novel 5′–3′ interaction architecture for trans-spliced mRNAs
Source: Nucleic Acids Res. 2018 Nov 22;47(3):1493–504. doi: 10.1093/nar/gky1187 (PMC6379680; doi:10.1093/nar/gky1187)
Supplement: Supplementary Data [file gky1187_supplemental_files.pdf]

Table S1. X-ray data collection statistics

	PABP1(J)	PABP1(J) + peptide
**Resolution range**	23.95 - 2.03 (2.103 - 2.03)	36.08 - 1.89 (1.958 - 1.89)
**Space group**	P 65	P 65
**Unit cell**	82.97 82.97 46.08 90 90 120	83.33 83.33 58.16 90 90 120
**Total reflections**	235407 (23284)	181863 (14218)
**Unique reflections**	11824 (1175)	18507 (1829)
**Multiplicity**	19.9 (19.8)	9.8 (7.8)
**Completeness (%)**	99.85 (99.91)	99.87 (99.84)
**Mean I/sigma(I)**	14.98 (1.67)	18.54 (1.32)
**Wilson B-factor**	39.23	42.68
**R-merge**	0.128 (1.821)	0.057 (1.5)
**R-meas**	0.1315 (1.868)	0.06019 (1.607)
**R-pim**	0.02967 (0.4187)	0.01918 (0.5737)
**CC1/2**	0.999 (0.683)	0.999 (0.586)
**CC***	1 (0.901)	1 (0.86)

Statistics for the highest-resolution shell are shown in parentheses.

1. Kabsch W (2010) XDS. *Acta Crystallogr D* 66:125-132.
2. Winter G (2010) xia2: an expert system for macromolecular crystallography data reduction. *Journal of Appl Crystallogr* 43:186-190.
3. Collaborative Computational Project Number 4 (1994) The CCP4 suite: programs for protein crystallography. *Acta Crystallogr D* 50(Pt 5):760-763.
4. McCoy AJ*, et al.* (2007) Phaser crystallographic software. *J Appl Crystallogr* 40:658-674.
5. Adams PD*, et al.* (2010) PHENIX: a comprehensive Python-based system for macromolecular structure solution. *Acta Crystallogr D* 66(Pt 2):213-221.
6. Terwilliger TC*, et al.* (2008) Iterative model building, structure refinement and density modification with the PHENIX AutoBuild wizard. *Acta Crystallogr D Biol Crystallogr* 64(Pt 1):61-69.
7. Emsley P, Lohkamp B, Scott WG, & Cowtan K (2010) Features and development of Coot. *Acta Crystallogr D Biol Crystallogr* 66(Pt 4):486-501.
8. Winn MD, Isupov MN, & Murshudov GN (2001) Use of TLS parameters to model anisotropic displacements in macromolecular refinement. *Acta Crystallogr D* 57(Pt 1):122-133.

Table S2. X-ray refinement statistics

	PAPB1(J)	PAPB1(J) + peptide
**Reflections used in refinement**	11819 (1175)	18505 (1831)
**Reflections used for R-free**	632 (56)	920 (79)
**R-work**	0.2015 (0.2819)	0.1956 (0.3268)
**R-free**	0.2251 (0.2644)	0.2307 (0.3492)
**CC(work)**	0.972 (0.789)	0.961 (0.793)
**CC(free)**	0.934 (0.829)	0.969 (0.746)
**No. of solvent**	72	50
**Protein residues**	159	155
**Solvent**	72	50
**RMS(bonds)**	0.002	0.007
**RMS(angles)**	0.43	0.86
**Ramachandran favored (%)**	99.35	98.83
**Rotamer outliers (%)**	0.00	1.25
**Clashscore**	3.11	1.03
**Average B-factor**	50.12	55.67
**PAPB1(J)**	49.93	54.49
**Peptide**		63.11
**solvent**	53.43	56.06
**Number of TLS groups**	2	4

Statistics for the highest-resolution shell are shown in parentheses.

Table S3. K_D estimates.

Technique	Proteins	K_{D1}/ K_{D2}	Figure
**SPR** heterogeneous model	eIF4E4(iv) + PABP1(J)	0.24×10^{-7}M / 6.3×10^{-7}M $^{+}$	5B
**SPR** homogeneous model	eIF4E4(iv) + PABP1(J)	1.6×10^{-7}M	5B
		**K_D**	
**Microscale thermophoresis**	eIF4E4(iv) + PABP1(J)	0.22×10^{-7}M*	5A
	eIF4E4(iv) + PABP1(J)F525A	5.5×10^{-7}M*	-
		**K_D**	
**Fluorescence anisotropy**	eIF4G3 + eIF4E4(v)::PABP1(J)	3.4×10^{-7}M	5C

*Average of three independent titrations.

$^{+}$The heterogeneous model provided the best fits to the SPR data (Supplementary Figure 7) and these values are accordingly taken to provide the most reliable indication of the affinity between eIF4E4(iv) and PABP1(J). K_{D1} and K_{D2} are the dissociation constants corresponding to the two states of eIF4E4(iv) in the model. The average of these two values (K_{av}) is 3.3×10^{-7}M.

Fig. S1. Synthesized peptides.

A: eIF4E4 PAM2 motif peptide (Fig. 3, Fig. *S4A*)

Sequence: **Ac-HHMNPNATEFMPGR-NH_2**

Calculated mass: 1678.7357 ; Observed mass: 1679.7497

HRMS deconvoluted spectrum

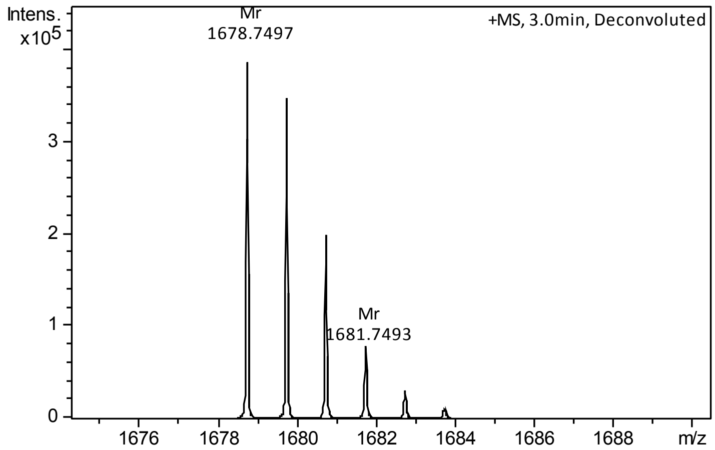

Analytical HPLC chromatogram

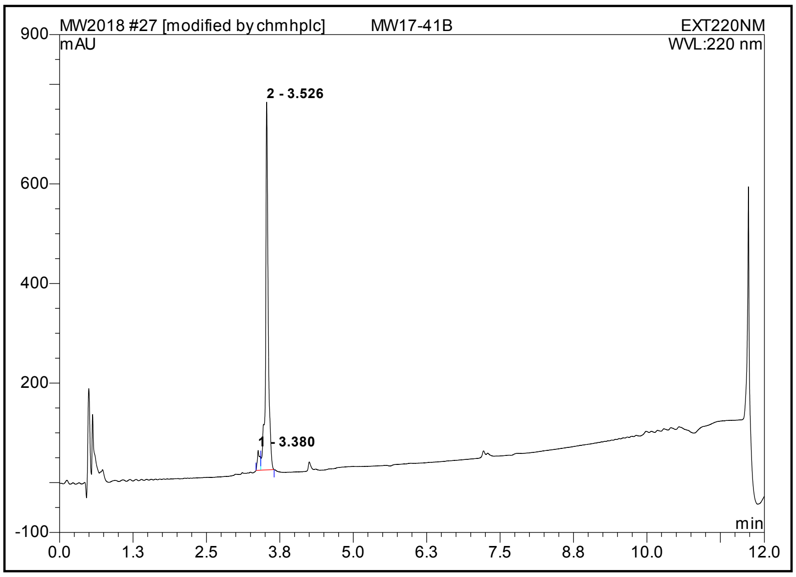

No.	Ret.Time min	Peak Name	Height mAU	Area mAU*min	Rel.Area %	Amount	Type
1	3.38	n.a.	39.870	1.959	4.93	n.a.	BM *
2	3.53	n.a.	738.756	37.810	95.07	n.a.	MB*
**Total:**			778.626	39.769	100.00	0.000	

Fig. S1. Synthesized peptides.

B: eIF4G3 dorsal face (eIF4E4-binding) peptide (Fig. S4*B*)

Sequence: **Ac-FTVEQIRSVRNNYLEPPYPGFSLDEVVR-NH_2**

Calculated mass: 3364.7094 ; Observed mass: 3364.7217

HRMS deconvoluted spectrum

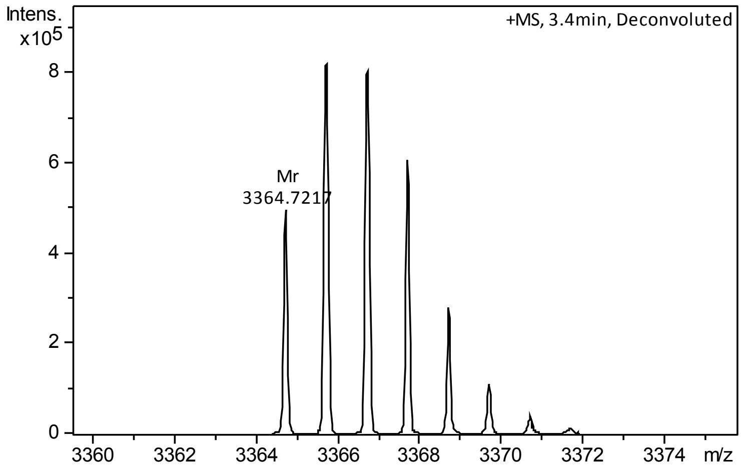

Analytical HPLC chromatogram

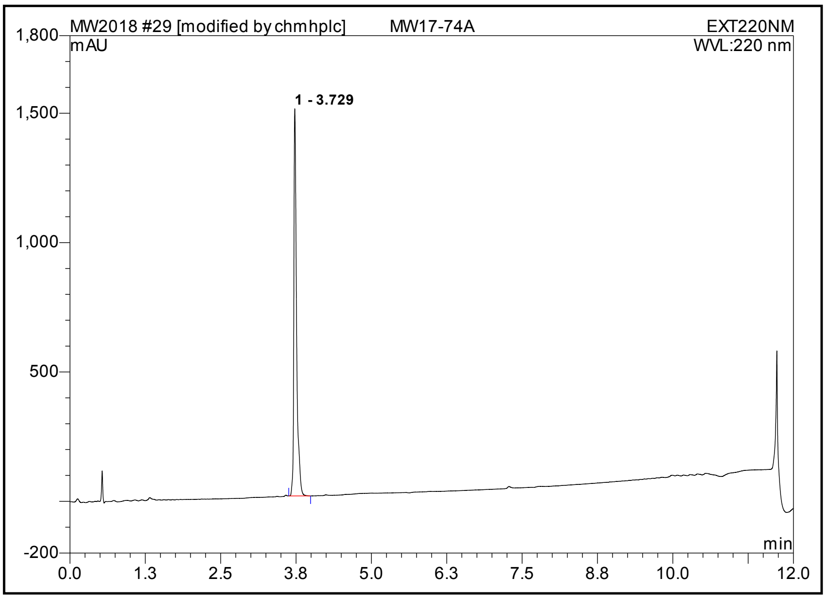

No.	Ret.Time min	Peak Name	Height mAU	Area mAU*min	Rel.Area %	Amount	Type
1	3.73	n.a.	1496.757	78.672	100.00	n.a.	BMB
Total:			1496.757	78.672	100.00	0.000	

Fig. S1. Synthesised peptides

C: Commercially sourced peptides used in mutational analysis of the *Leishmania* eIF4E4-PAB1P binding interface.

**eIF4E4$_{138\text{-}151}$ WT**	HH**M**NP**N**AT**EFM**PGR
**eIF4E4$_{138\text{-}151}$ M140A**	HH**A**NP**N**AT**EFM**PGR
**eIF4E4$_{138\text{-}151}$ N143A**	HH**M**NP**A**AT**EF**MPGR
**eIF4E4$_{138\text{-}151}$ E146A**	HH**M**NP**N**AT**AF**MPGR
**eIF4E4$_{138\text{-}151}$ F147A**	HH**M**NP**N**AT**EA**MPGR
**eIF4E4$_{138\text{-}151}$ M148A**	HH**M**NP**N**AT**EFA**PGR
**eIF4E4$_{138\text{-}151}$ NEFM→A**	HH**M**NP**A**AT**AAA**PGR

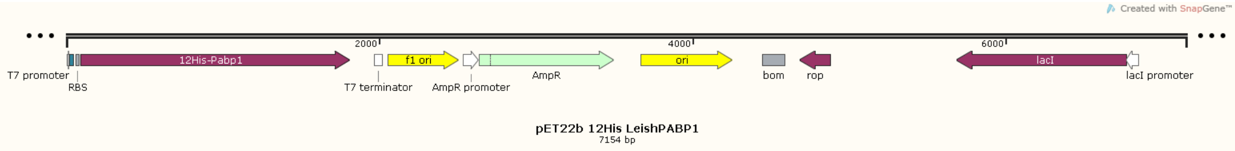

IF4E4 domains:		
	pET22b Leish eIF4E4-sector-4-12His	
	pET22b Leish eIF4E4-12His	
Pabp1 domains:		
	pET22b 12His-LeishPabp1 full length	pET22b 12His-LeishPabp1-domain I
	pET22b 12His-LeishPabp1-domain A	pET22b 12His-LeishPabp1-domain J
	pET22b 12His-LeishPabp1-domain B	pET22b 12His-LeishPabp1-domain K
	pET22b 12His-LeishPabp1-domain C	pET22b 12His-LeishPabp1-domain M
	pET22b 12His-LeishPabp1-domain D	pET22b 12His-LeishPabp1-domain N
	pET22b 12His-LeishPabp1-domain E	pET22b 12His-LeishPabp1-domain O
	pET22b 12His-LeishPabp1-domain F	pET22b 12His-LeishPabp1dA
	pET22b 12His-LeishPabp1-domain G	pET22b 12His-LeishPabp1dC
	pET22b 12His-LeishPabp1-domain H	pET22b 12His-LeishPabp1dJ

eIF4G3 expression:	
	pET22b Leish eIF4G3-12His full length

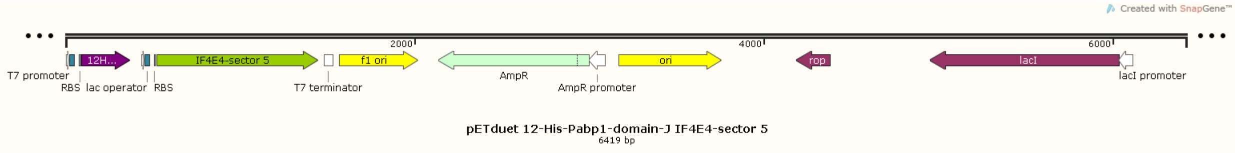

Co-expression of Pabp1 and eIF4E4 domains:	
	pETduet 12His-Pabp1-domain-J eIF4E4 ful length
	pETduet 12His-Pabp1-domain-J eIF4E4-sector 5
	pETduet Pabp1-domain-J eIF4E4-sector 5
	pETduet Pabp1-domain-G eIF4E4-sector 5

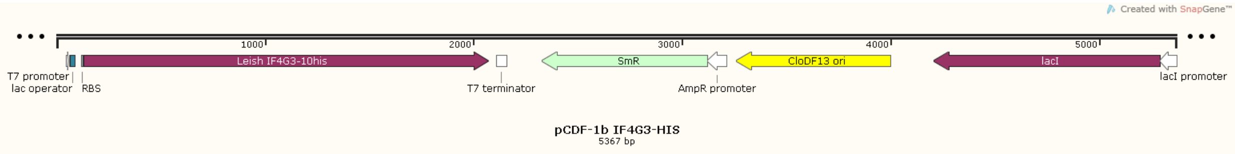

co-expression of eIF4G3 or Pabp1 (used together with pETduet or pET22b vectors):	
	pCDF-1b Leish eIF4G3-12His
	pCDF-1b Leish eIF4G3
	pCDF-1b Leish Pabp1

Fig. S2 Scheme of the co-expression plasmids used in *E.coli* as the starting point for generating *Leishmania* protein complexes.

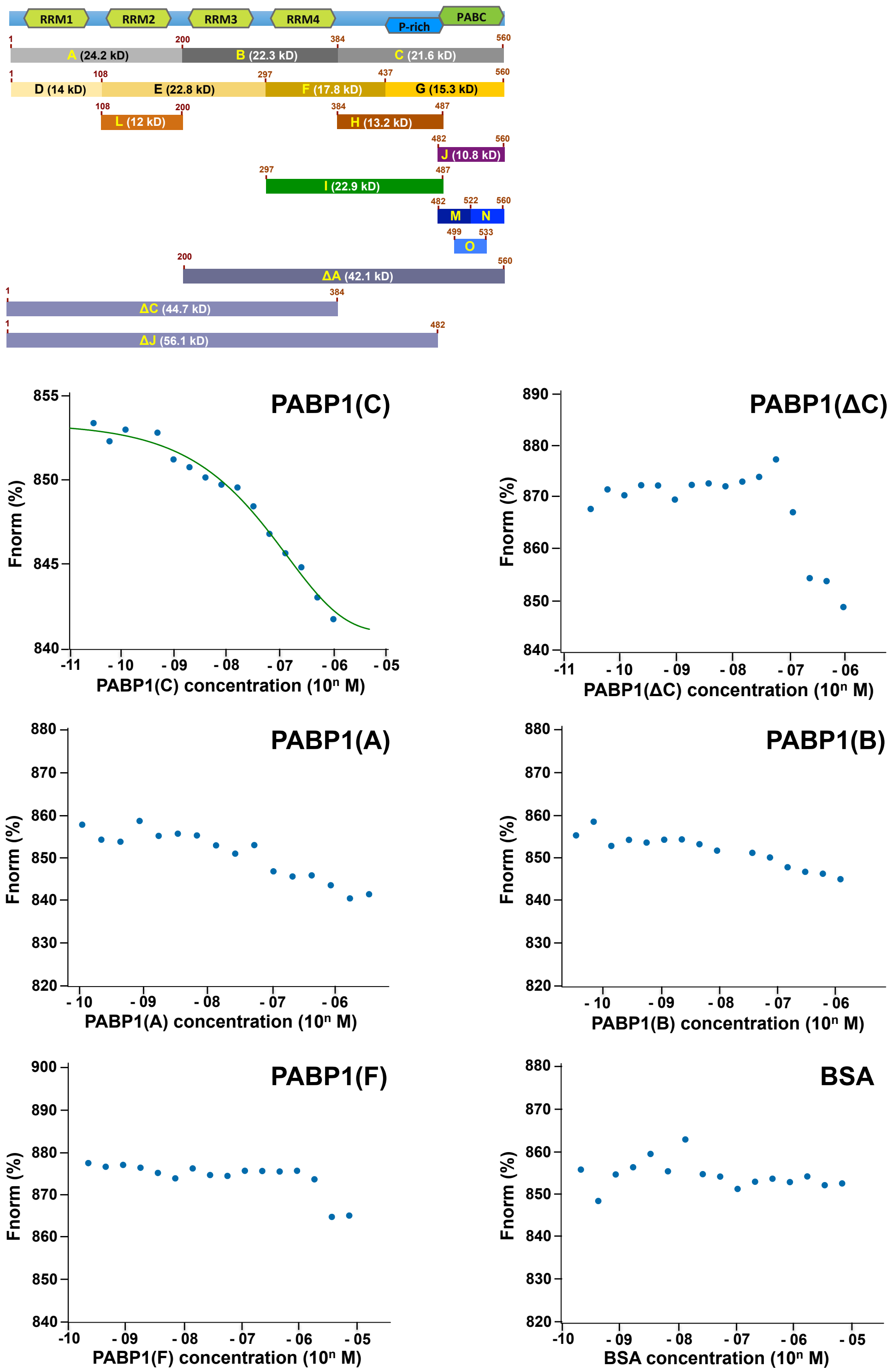

Fig. S3 Microscale thermophoresis screening of PABP1 protein sections for binding to eIF4E4(iv). Multiple examples of the titrations underpinning the data summarised in Fig. 1*C* are shown, collectively covering the complete sequence of PABP1. A control experiment, in which bovine serum albumin (BSA) was substituted for PABP1, is also shown. Aggregation of the PABP1(ΔC) protein at higher concentrations caused a distortion of the titration curve.

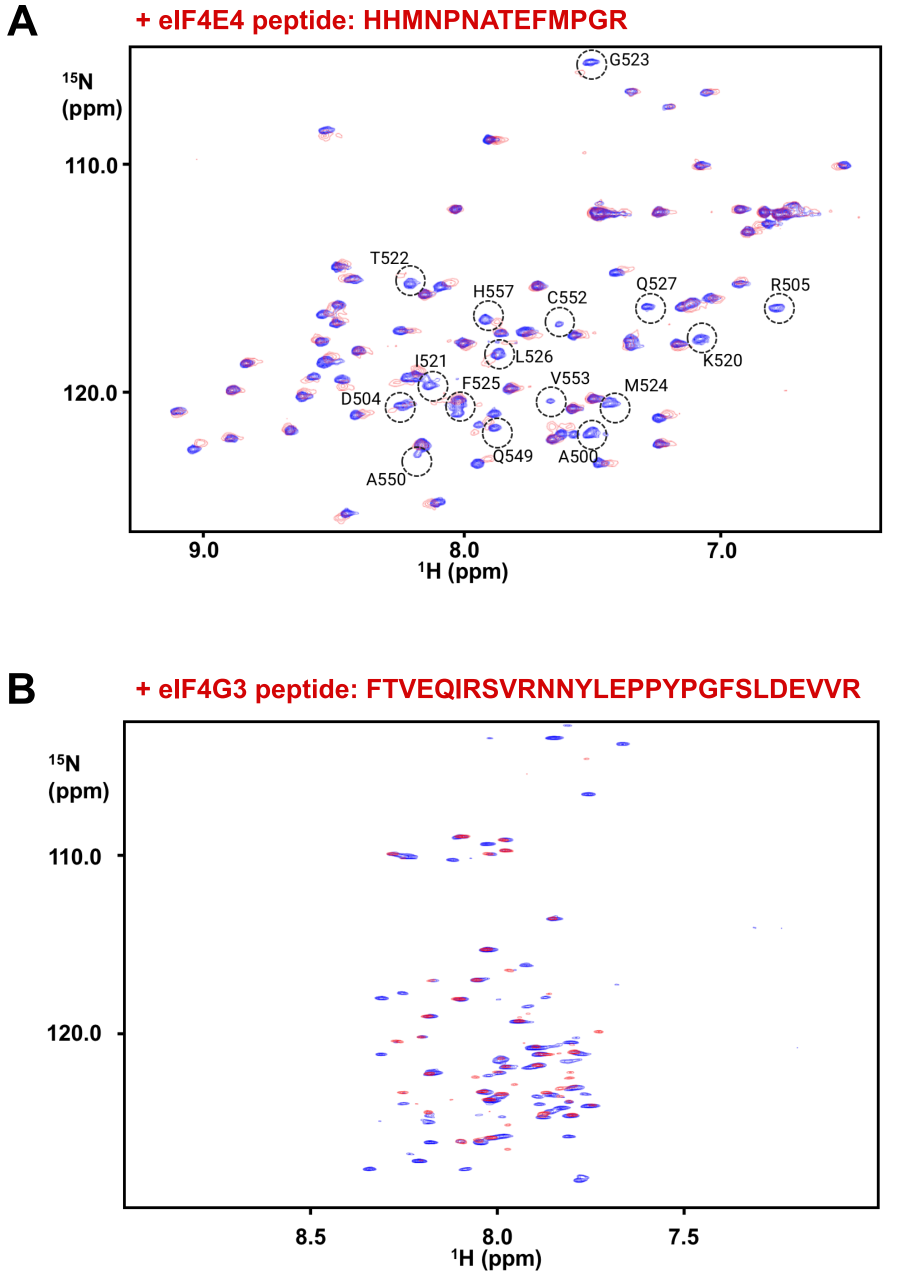

Fig. S4 (***A***) Triple resonance (CBCANNH, CBCA(CO)NNH) assignments were performed and titrations of ^{13}C-^{15}N- PABP1(J) vs unlabeled eIF4E4 peptide (sequence shown) were used to generate assigned ^{1}H-^{15}N- HSQC spectra (free ^{13}C-^{15}N- PABP1(J) in blue; bound to eIF4E4 peptide in red).

(***B***) ^{1}H-^{15}N- HSQC spectra of the heterodimeric complex formed by ^{15}N-,^{13}C- labeled PABP1(J) and ^{15}N,^{13}C- labeled eIF4E4(v) [in blue: just this heterodimer; in red: upon addition of a synthetic 28mer peptide (amino acid sequence given in red) that includes the eIF4G3 motif that binds the eIF4E4 dorsal face].

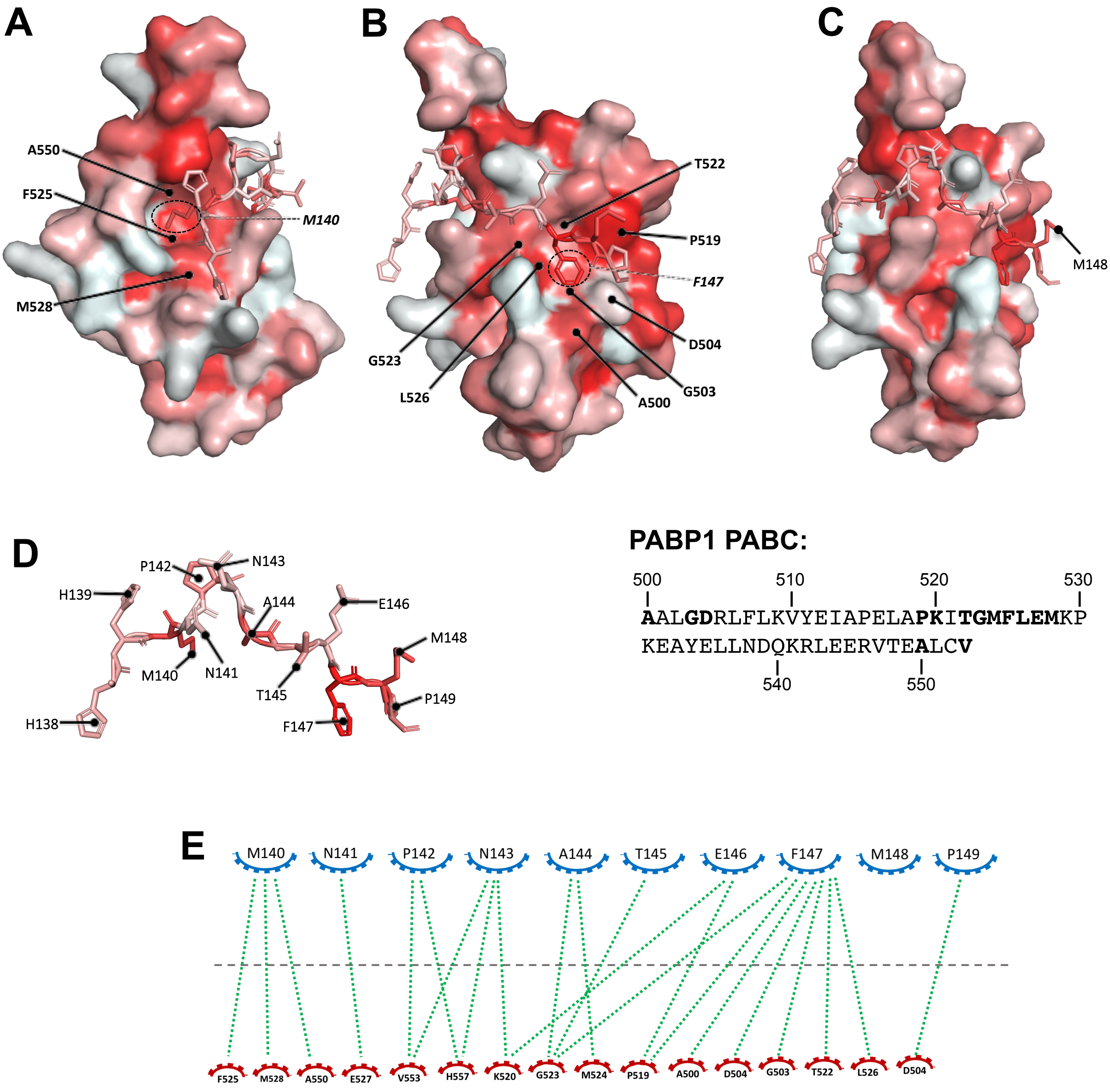

Fig. S5 Hydrophobicity plots for the complex between PABP1(J) and the eIF4E4 PAM2 peptide. Three different orientations (**A-C**) of the complex (compare Fig. 3C) are shown. Panel **D** shows, on the left, the backbone structure of the eIF4E4 peptide (presented separately) and, on the right, the PABC sequence from PABP1. The intensity of the red colour corresponds to the degree of hydrophobicity calculated for the respective regions of the molecular structures. Panel **E** is a LIGPLOT (*see SI Text*) representation of the respective atomic interactions between the two protein molecules.

A

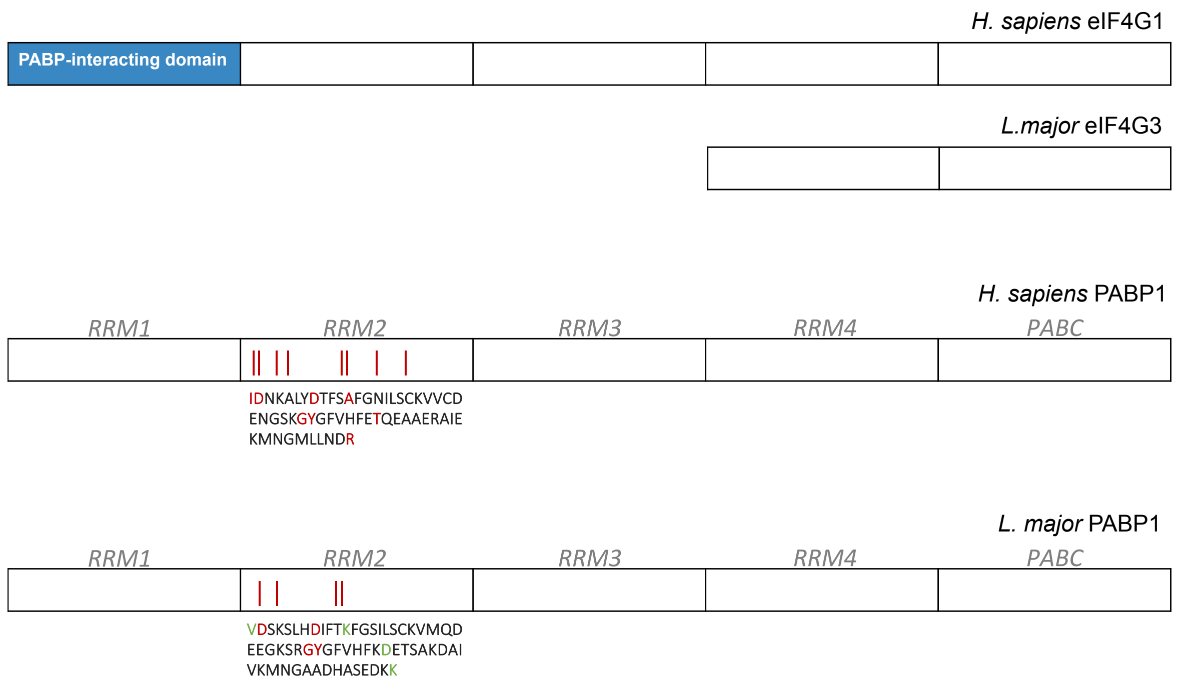

B

```
                 26                                        67
L.major eIF4E3:  AVAKPPSTQPATKLSAAAEPFVPGGPKQMSATSTHVDPKATTE
L.major eIF4E4:  PTRFSPATVPRHHMNPNATEFMPGRRNGPDGGLEA-LPTSTAD
                127                                        168
```

Fig. S6
(***A***) Overall structure (and motif) comparisons for eIF4G and PABP1 proteins from *H.sapiens* and *Leishmania*. These highlight the absence of recognised (canonical) PABP1-binding motif residues in *L.major* eIF4G3 and of key residues in *L.major* PABP1 that are known to be important for eIF4G binding in *H.sapiens* PABP1.

(***B***) The PAM2 sequence in the N-terminal extension of *L.major* eIF4E4 is only partially shared by the N-terminal region of eIF4E3. Identical amino acids are highlighted in red; non-identical residues belonging to the same amino-acid-class are highlighted in blue.

C

```
PABP1: LPPITPQELESMSPQEQRAALGDRLFLKVYEIAPELAPKITGMFLEMKPK
PABP2: QGQNLAAVLANLNPEQQKNVLGERLYSYIVRSHPSVAAKITGMLLEMDNA
PABP3: QDGVDMNYLSTLSPEQQKNYLGELLYSRILPLESSNAAKITGMLLEMSRE

PABP1: EAYELLNDQKRLEERVTEALCVLKAHQTA
PABP2: EILNMLDSPTMLDSKIAEAQDVLNRHMSV
PABP3: EIFEILADHFALLSKIQEANAVLQQHTGN
```

D

```
eIF4E4
L.major            PAVAARSVPTRFSPATVP----RHHMNPNATEFMPGRRNG
L.panamensis       -------------------------MSPNATEFVPGRSNG
L.guyanensis       PAVAARSVPTRFSPATVP----R-HMSPNATEFVPGRSNG
L.braziliensis     PAVAARSVPTRFSPATVP----R-HMNPNATEFVPGRSNG
L.mexicana        --------------------------MNPNATEFMPGRRNG
L.infantum        --------------------------MNPNATEFMPGRRNG
T.brucei          --PVSTHVIPTRMSPVHAPS--AAFHMSPNAVSYVPRGA--
T.theileri        --PTAALRFPTRMSPMHAPFPPVSSTMNPDAKDFIPHLS--
T.cruzi Brener    --PTTALRLPTRMSPMHAPFPSVSISMNPNATDFVPHLT—

PABP1
L. major          KITGMFLEMKPKEAYELLNDQKRLEERVTEALCVLKAHQTA
L. panamensis     KITGMFLEMNPKEAYELLNDQKRLEERVTEALCVLKAHQTV
L. guyanensis     KITGMFLEMNPKEAYELLNDQKRLEERVTEALCVLKAHQTA
L. braziliensis   KITGMFLEMNPKEAYELLNDQKRLEERVTEALCVLKAHQTV
L. mexicana       KITGMFLEMKPKEAYELLNDQKRLEDRVTEALCVLKAHQTT
L. infantum       KITGMFLEMKPKEAYELLNDQKRLEERVTEALCVLKAHQTA

T. brucei         KITGMFLEMNPKEALALLSNPKLMHEKVTEALCVLKVHASS
T. theileri       KITGMFLEMDLKEAFTLLYNQKLLHEKVTEALCVLKAHGTT
T. cruzi Brener   KITGMFLEMDLKEAFTLLTNQRLLQEKVIEALCVLKAHEST
```

Fig. S6

(***C***) Comparison of the PABC domains of *L.major* PABP1, 2 and 3. Identical residues are in bold, amino acids belonging to the same category are in blue.

(***D***) Comparison of eIF4E4 (PAM2) and PABP1 PABC sequences of other trypanosomatid species. It is evident that the PABP1 sequences across the respective species are much more similar to each other than are the sequences of PABP1, 2 and 3 within one species, in this case *L.major*.

A

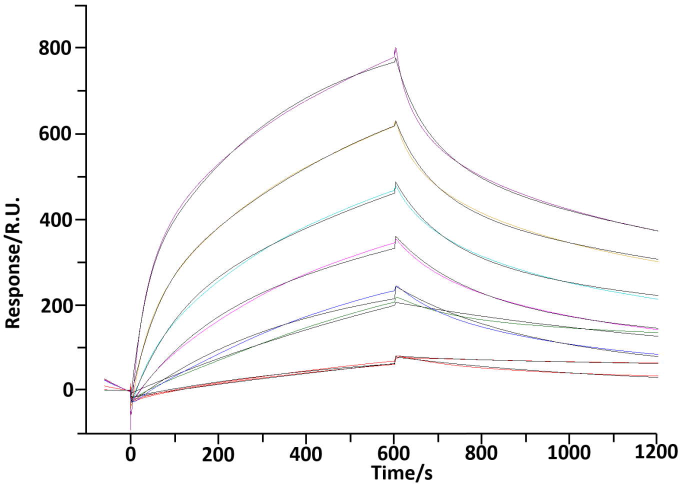

B

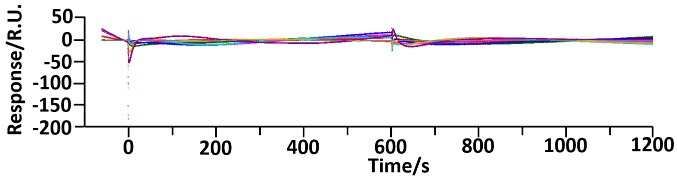

Fig. S7

Curve fitting for the SPR data shown in Figure 5B. Here we have applied a heterogeneous ligand model, which is recommended as an approximation for binding to an amine-coupled protein, since the latter can be immobilized in different orientations. This model assumes that the ligand [here PABP1(J)] binds to two alternative states of the immobilized protein [here eIF4E4(iv)], each with its own association and dissociation rates. The fitting lines (**A**) are shown in black; the residuals (**B**) are small, indicating that this model generates good fits. The Tables present the parameters used to achieve the fits. We also compared a simpler, single binding state model, which generated less convincing fits (data not shown).

C

Report | Residuals | Parameters

Curve	ka1 (1/Ms)	kd1 (1/s)	KD1 (M)	ka2 (1/Ms)	kd2 (1/s)	KD2 (M)	Rmax1 (RU)	Rmax2 (RU)	Conc (M)	tc	Flow (ul/min)	kt (RU/Ms)	RI (RU)	Chi² (RU²)	U-value
	1929	5.748E-4	2.980E-7	3.221E+4	0.01853	5.753E-7				4.664E+6				33.7	N/A
Cycle: 77 0.0015 mg/ml							5978	1029	2.329E-8		10.00	1.005E+7	-18.24		
Cycle: 78 0.0015 mg/ml							441.3	2818	2.329E-8		10.00	1.005E+7	-16.27		
Cycle: 79 0.003 mg/ml							1291	5747	4.658E-8		10.00	1.005E+7	-7.436		
Cycle: 80 0.006 mg/ml							643.2	1866	9.317E-8		10.00	1.005E+7	-30.22		
Cycle: 81 0.0121 mg/ml							1073	1075	1.879E-7		10.00	1.005E+7	-30.78		
Cycle: 82 0.0242 mg/ml							986.2	657.9	3.758E-7		10.00	1.005E+7	-30.37		
Cycle: 83 0.0484 mg/ml							824.8	465.3	7.516E-7		10.00	1.005E+7	-18.06		
Cycle: 84 0.0968 mg/ml							681.2	422.4	1.503E-6		10.00	1.005E+7	-18.64		

Report | Residuals | Parameters

Curve	ka1 (1/Ms)	SE(ka1)	kd1 (1/s)	SE(kd1)	ka2 (1/Ms)	SE(ka2)	kd2 (1/s)	SE(kd2)	Rmax1 (RU)	SE(Rmax1)	Rmax2 (RU)	SE(Rmax2)	Conc (M)	tc	SE(tc)	f (ul/min)	RI (RU)	SE(RI)
	1929	7.2	5.748E-4	2.1E-6	3.221E+4	2.6E+2	0.01853	1.2E-4						4.664E+6	1.4E+4			
Cycle: 77 0.0015 mg/ml									5978	37	1029	27	2.329E-08			10	-18.2	0.14
Cycle: 78 0.0015 mg/ml									441.3	35	2818	21	2.329E-08			10	-16.3	0.10
Cycle: 79 0.003 mg/ml									1291	96	5747	60	4.658E-08			10	-7.4	0.13
Cycle: 80 0.006 mg/ml									643.2	7.3	1866	6.9	9.317E-08			10	-30.2	0.15
Cycle: 81 0.0121 mg/ml									1073	3.9	1075	3.4	1.879E-07			10	-30.8	0.16
Cycle: 82 0.0242 mg/ml									986.2	2.8	657.9	1.7	3.758E-07			10	-30.4	0.20
Cycle: 83 0.0484 mg/ml									824.8	1.8	465.3	1.1	7.516E-07			10	-18.1	0.26
Cycle: 84 0.0968 mg/ml									681.2	1.0	422.4	0.84	1.503E-06			10	-18.6	0.35

ka1= association rate constant
kd1= dissociation rate constant
KD1= equilibrium dissociation constant
ka2= association rate constant
kd2= dissociation rate constant
KD2= equilibrium dissociation constant
Rmax1= Maximum response
Rmax2= Maximum response
Con= Concentration
kt= Mass transfer constant
RI= Refractive Index
RU= Resonance units
Chi2=Chi-squared
U-value= Uniqueness correlation parameter

SE(ka1)= Standard error of the association rate constant
SE(kd1)= Standard error of the dissociation rate constant
SE(ka2)= Standard error of the association rate constant
SE(kd2)= Standard error Standard error of the dissociation rate constant
SE(Rmax1)= Standard error of the maximum response
SE(Rmax2)= Standard error of the maximum response
tc= flow rate-independent component
SE(tc)= Standard error of the flow rate-independent component
SE(RI)= Standard error of the refractive index

Fig. S7
(**C**) Parameters used in the fitting of the heterogeneous binding model to the SPR data (see panel A).

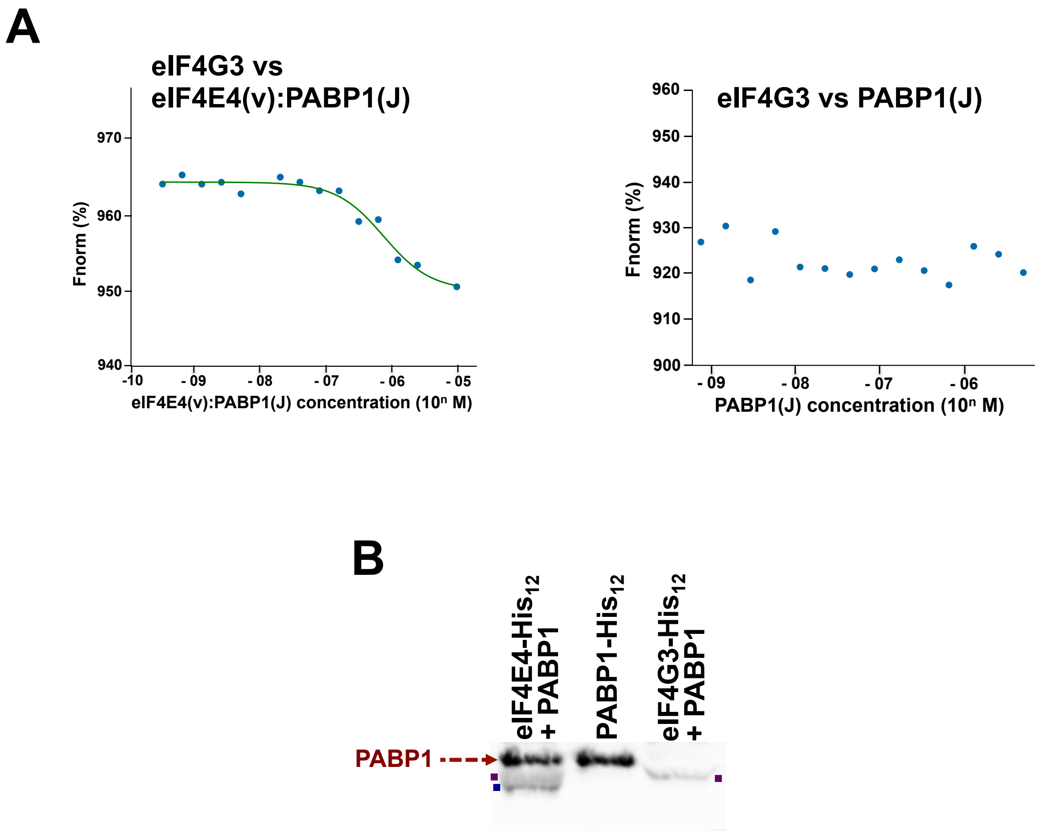

Fig. S8. eIF4G3 binds to eIF4E4 via dorsal-face motifs. (***A***) Microscale thermophoresis reveals binding between Alexa-647-labelled-eIF4G3 and eIF4E4(v):PABP1(J) but not between Alexa-647-labelled-eIF4G3 and PABP1(J) alone. The binding of PABP1(J) hinders aggregation of eIF4E4(v). We observed evidence of ligand-dependent quenching of the fluor attached to eIF4G3, which means that an accurate K_D value cannot be calculated directly from these data. (***B***) Pull-down experiments performed with lysates from *E.coli* expression strains producing eIF4E4-His_{12} + PABP1, PABP1-His_{12} alone, and eIF4G3-His_{12} + PABP1 (Fig. S4). In each case, Lysates were incubated with a tetradentate chelating agarose resin charged with divalent cobalt Ions and, after column washing, the His_{12}-tagged proteins were eluted using imidazole. The eluates were subjected to Western blotting using rabbit anti-*L.major*-PABP1 serum. We could detect no co-eluted PABP1 when eIF4G3 was His_{12}-tagged. There was a low level of cross-recognition of *E.coli* proteins by the antibody (purple and blue dots).
